# Novel roles of ER stress in repressing neural activity and seizures through Mdm2- and p53-dependent protein translation

**DOI:** 10.1371/journal.pgen.1008364

**Published:** 2019-09-26

**Authors:** Dai-Chi Liu, Daphne E. Eagleman, Nien-Pei Tsai

**Affiliations:** 1 Neuroscience Program, University of Illinois at Urbana-Champaign, Urbana, Illinois, United States of America; 2 Department of Molecular and Integrative Physiology, School of Molecular and Cellular Biology, University of Illinois at Urbana-Champaign, Urbana, Illinois, United States of America; Columbia University Medical Center, UNITED STATES

## Abstract

Seizures can induce endoplasmic reticulum (ER) stress, and sustained ER stress contributes to neuronal death after epileptic seizures. Despite the recent debate on whether inhibiting ER stress can reduce neuronal death after seizures, whether and how ER stress impacts neural activity and seizures remain unclear. In this study, we discovered that the acute ER stress response functions to repress neural activity through a protein translation-dependent mechanism. We found that inducing ER stress promotes the expression and distribution of murine double minute-2 (Mdm2) in the nucleus, leading to ubiquitination and down-regulation of the tumor suppressor p53. Reduction of p53 subsequently maintains protein translation, before the onset of translational repression seen during the latter phase of the ER stress response. Disruption of Mdm2 in an *Mdm2* conditional knockdown (cKD) mouse model impairs ER stress-induced p53 down-regulation, protein translation, and reduction of neural activity and seizure severity. Importantly, these defects in *Mdm2* cKD mice were restored by both pharmacological and genetic inhibition of p53 to mimic the inactivation of p53 seen during ER stress. Altogether, our study uncovered a novel mechanism by which neurons respond to acute ER stress. Further, this mechanism plays a beneficial role in reducing neural activity and seizure severity. These findings caution against inhibition of ER stress as a neuroprotective strategy for seizures, epilepsies, and other pathological conditions associated with excessive neural activity.

## Author summary

One-third of epilepsy patients respond poorly to current anti-epileptic drugs. Thus, there is an urgent need to characterize cellular behavior during seizures, and the corresponding molecular mechanisms in order to develop better therapies. Seizures are known to induce ER stress but how the ER stress response functions to modulate seizure activity is unknown. Our study provides evidence to demonstrate a novel and beneficial role for the ER stress response in reducing neural activity and seizure severity. Mechanistically, we found that these beneficial effects are mediated by elevated protein translation, which is triggered by the activation of Mdm2-p53 signaling, during the early ER stress response. Our findings suggest that therapeutic attempts to reduce ER stress in epilepsies may result in worsening seizure activity and therefore caution against inhibition of ER stress as a neuroprotective strategy for epilepsies.

## Introduction

A seizure is an uncontrolled electrical disturbance in the brain. Recurrent and spontaneous seizures lead to epilepsy, a pathological condition affecting 50 million people worldwide. Despite the development of antiepileptic drugs that seek to raise the seizure threshold, one-third of epilepsy patients either respond poorly to these drugs or remain drug-resistant [1, 2]. To improve therapeutic outcomes, it is necessary to have a substantial understanding of the molecular and cellular mechanisms which occur during seizure onset.

In addition to eliciting profound neural activities and behavioral changes, seizures result in the excessive release of neurotransmitters, such as glutamate, that can cause excitotoxicity in the brain [3]. Excitotoxicity is particularly apparent in chronic epileptic brains, where neuronal degeneration and cell damage have been observed [4–6]. Among the cellular mechanisms that contribute to excitotoxicity-induced cell damage, endoplasmic reticulum (ER) stress has gained much attention [7, 8]. ER stress is caused by disturbances in a cell’s growth environment, which can include viral infection, nutrient starvation, accumulation of unfolded proteins, and excitotoxicity, among many [9–12]. The cellular response to ER stress is comprised of a set of evolutionarily conserved mechanisms that serve to help the cell adapt to and remove disturbances. When the attempts to cope with the disturbances fail or when the disturbances last for an extended period of time, the ER stress response can trigger cell death. Although ER stress has been observed and suggested to contribute to cell death in various brain regions after chronic seizures or epilepsies [5], a recent study has also proposed that ER stress can be crucial for neuronal survival after seizures [3]. Despite this controversy, it remains unknown whether and how the ER stress response might affect or modulate the hyperexcitability that occurs during seizure onset.

In our current study, we utilized a multi-electrode array (MEA) recording system with cultured primary cortical neurons and a kainic acid-induced seizure model in mice to reveal a novel and beneficial role for the acute ER stress response in reducing neural activity and seizure severity. We subsequently showed that this phenomenon is dependent on protein translation triggered by ubiquitination and down-regulation of tumor suppressor p53. The ubiquitination of p53 is mediated by the ubiquitin E3 ligase Mdm2, whose expression is transcriptionally elevated upon induction of ER stress. Although Mdm2-p53 signaling has been shown to participate in cellular stress-induced apoptosis [4, 13], our study supports the previous study [3] and demonstrates a beneficial role for ER stress response in neural excitability homeostasis after seizures. Our findings also indicate that any attempts to ameliorate seizure-induced cell death by inhibiting ER stress may actually worsen seizure severity by diminishing the homeostatic effect on neural activity induced upon ER stress. Both the positive and negative consequences of ER stress should be taken into consideration when developing and testing the next generation of seizure therapies.

## Results

### Acute ER stress reduces seizure severity

**Fig 1:**
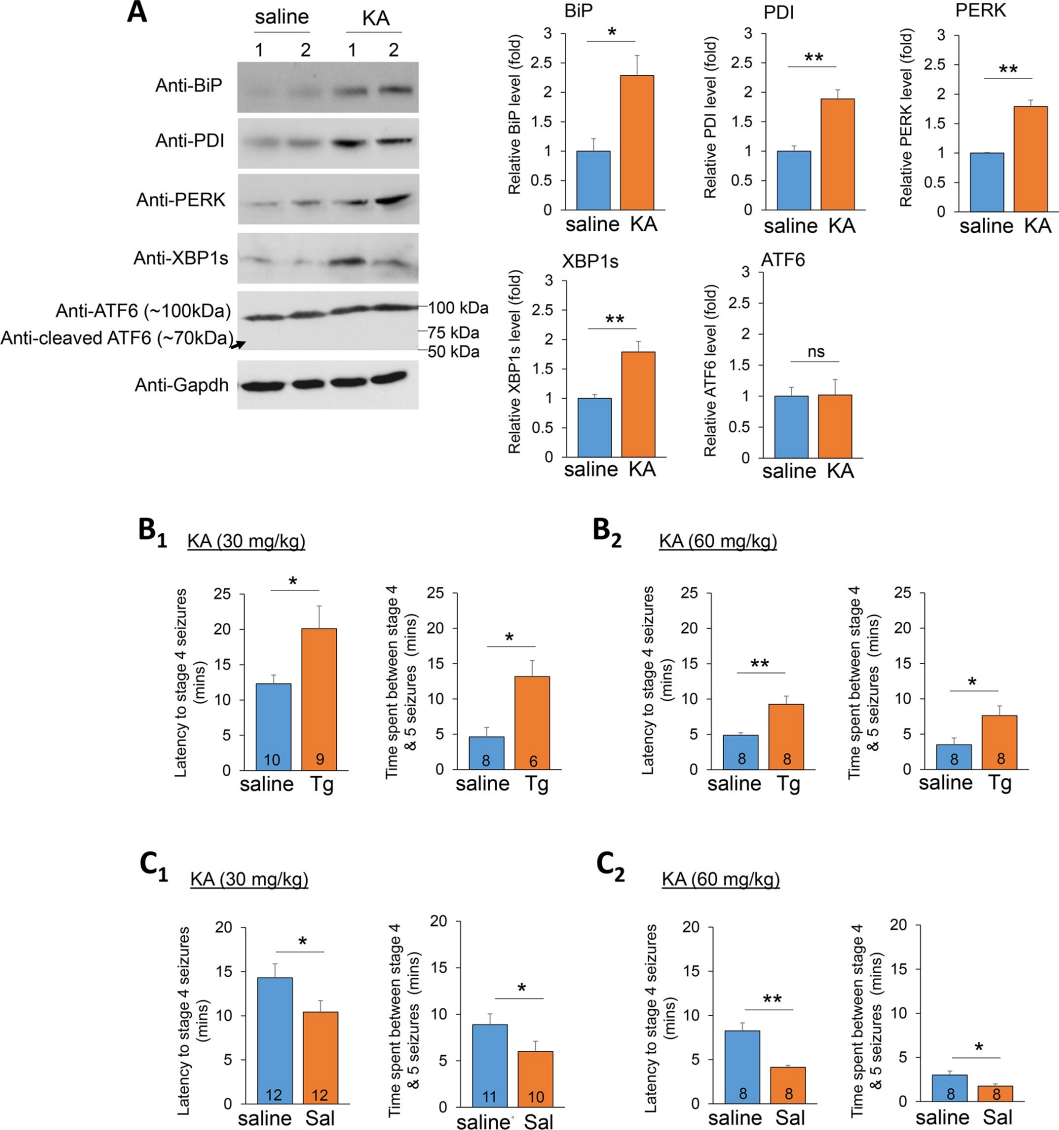
Acute ER stress reduces seizure severity. (A) Representative western blots of BiP, PDI, PERK, XBP1s, ATF6 and Gapdh from total brain lysate of two pairs of 3-week old WT mice intraperitoneally injected with saline or kainic acid (KA, 60 mg/kg). The arrow indicates the predicted position for cleaved ATF6. Quantification is shown on the right (n = 4). (B) Quantification of latency to stage 4 seizures, and the time spent between stage 4 to 5 seizures from 3-weeks old WT mice intraperitoneally injected with saline or Thapsigargin (Tg, 2 mg/kg) for 3 hours followed by injections with KA (B_1_: 30 mg/kg, or B_2_: 60 mg/kg). The number of mice used in each condition is shown on the bottom of each bar. (C) Quantification of latency to stage 4 seizures, and the time spent between stage 4 to 5 seizures from 3-weeks old WT mice intraperitoneally injected with saline or Salubrinal (Sal, 2 mg/kg) for 3 hours followed by injections with KA (C_1_: 30 mg/kg, or C_2_: 60 mg/kg). The number of mice used in each condition is shown on the bottom of each bar. Student’s *t*-test was used. Data are represented as mean ± SEM with *P<0.05, **P<0.01, ns: non-significant.

It has been previously suggested that ER stress contributes to seizure-induced neuronal damage [5, 14]. However, it is unclear whether ER stress plays a role in counteracting or exacerbating the insult, such as hyperactivity, observed during the onset of seizures. To answer this question, we employed a kainic acid-induced seizure model in C57BL/6J mice to determine whether ER stress affects seizure severity. We first aimed to replicate the observation that kainic acid-induced seizures are accompanied by ER stress in the brain, as previously reported [3]. As shown in Fig 1A, wild-type (WT) mice intraperitoneally injected with kainic acid (60 mg/kg), who showed apparent seizure activity within 30 minutes, exhibited significantly elevated expression of four ER stress markers in the brain when compared to the mice injected with saline only: binding immunoglobulin protein (BiP), protein disulfide isomerase (PDI), protein kinase RNA-like endoplasmic reticulum kinase (PERK), and the spliced isoform of X-box binding protein 1 (XBP1s). Interestingly, another ER stress indicator, the cleavage of activating transcription factor 6 (ATF6), detected by a previously validated antibody [15], was not observed after seizures. These results suggest an acute induction of selective ER stress pathways upon the onset of seizures.

To determine whether acute ER stress can affect neuronal hyperactivity during seizures, we next asked whether inducing ER stress prior to seizure onset could affect seizure severity. To this end, we intraperitoneally injected littermate mice with saline or Thapsigargin (Tg, 2 mg/kg) [16], a commonly used drug which induces ER stress through inhibition of ER Ca^2+^ ATPase, for three hours. This dosage, administration route, and treatment duration were based upon a previous study that demonstrated successful induction of ER stress in the brain [16]. Three hours after injecting saline or Tg, the mice were injected with kainic acid at 30 mg/kg or 60 mg/kg. The use of these relatively high dosages of kainic acid is due to the fact that mice of C57BL/6 background are relatively resistant to kainic acid-induced seizures [17–20]. Immediately following injections, ER stress markers and seizure behavior were quantified. As shown, the mice who received Tg showed an elevation of ER stress markers (S1 Fig) and a significant delay in the onset of the stage 4 seizures (rearing and falling) and an extended time between stage 4 and stage 5 seizures (tonic-clonic seizures), as compared to the mice receiving saline (Fig 1B). The same results were also observed with older, 12-weeks old, mice (S2 Fig), suggesting the effect is likely independent of development stages. To determine whether a similar effect can be seen after a chronic induction of ER stress, we injected littermate mice with saline or Tg at a lower dose (0.5 mg/kg) for 48 hours [21]. As shown (S3 Fig), a significant delay in seizure activities was also observed after the chronic induction of ER stress. These results indicate that the effect of ER stress on reducing seizure severity is likely a long-lasting event.

We then asked whether this reduction in seizure severity upon induction of ER stress occurred as a result of activation of ER stress response pathways. To this end, we employed an inhibitor of the ER stress response, Salubrinal (2 mg/kg), which acts through inhibition of eukaryotic translation initiation factor 2α (eIF2α) dephosphorylation [22]. As shown, mice receiving Salubrinal three hours before kainic acid showed a reduction of ER stress markers (S1 Fig) and an earlier onset of stage 4 seizures and a reduction in the time spent between stage 4 and stage 5 seizures, when compared to mice receiving saline, following injections of kainic acid at 30 mg/kg or 60 mg/kg (Fig 1C). Together, our results indicate that inducing the acute ER stress response reduces seizure severity in mice.

### Acute ER stress reduces neural activity through a protein translation-dependent mechanism

**Fig 2:**
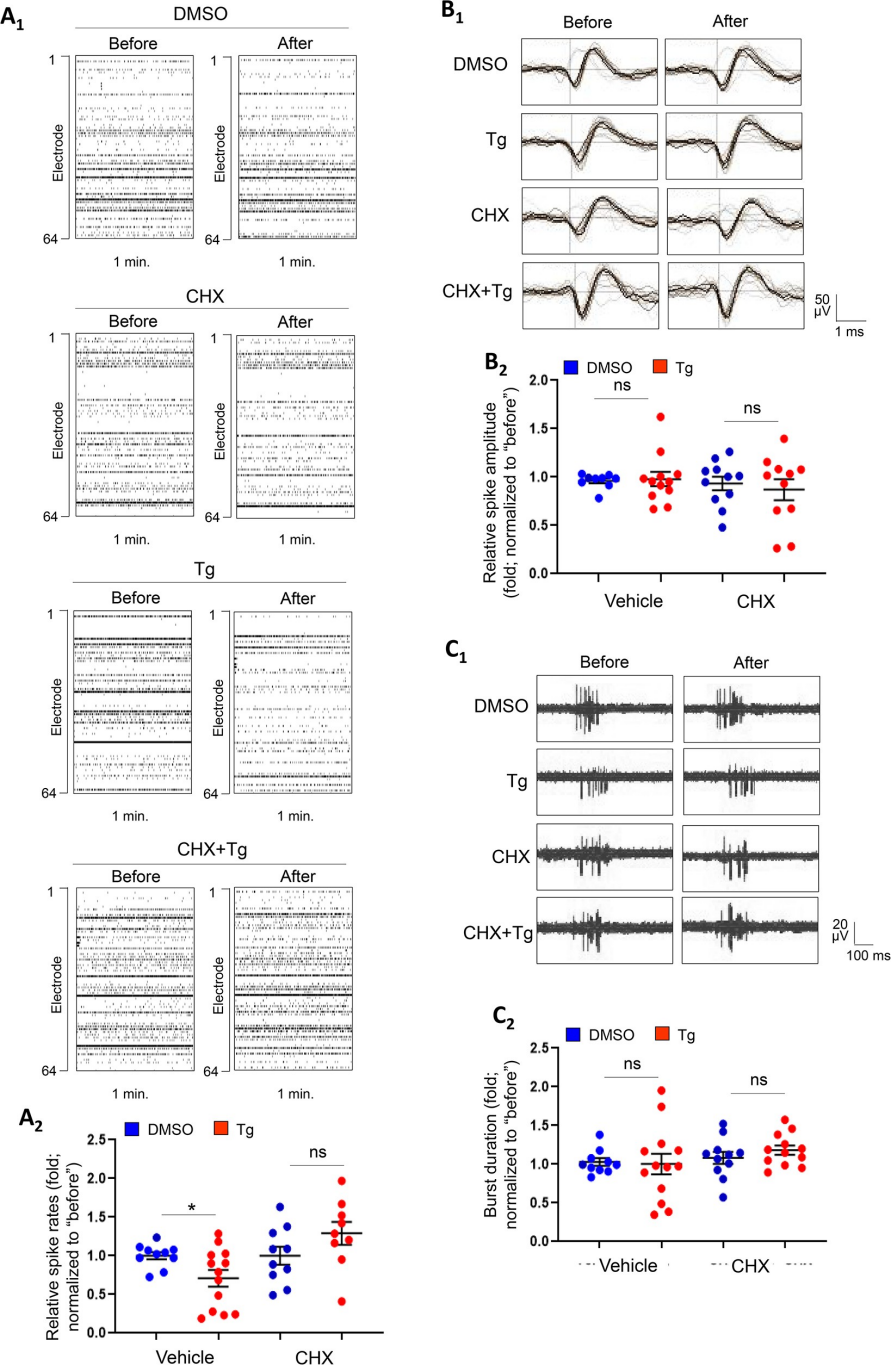
Acute ER stress induces protein translation-dependent reduction of neural network activity. (A_1_) Raster plots of spontaneous spikes from representative 1-min recordings of WT cortical neuron cultures treated with vehicle (DMSO), Thapsigargin (Tg, 1 μM), cycloheximide (CHX, 60 μM) or CHX+Tg for 1 h at DIV14. (A_2_) Quantification of relative spontaneous spike rates by comparing ‘after treatment’ to ‘before treatment’ of the same cultures during the 15-min recordings. (B_1_) Representative average traces of spike amplitude from 1-min recordings of WT cortical neuron cultures treated with DMSO, Tg, CHX, or CHX+Tg for 1 h at DIV14. In the traces, the black lines represent the average of all the spikes within representative 1-min recordings. Traces are from the same designated electrodes before and after treatments. (B_2_) Quantification of average spontaneous spike amplitude by comparing ‘after treatment’ to ‘before treatment’ during the 15-min recordings of the same cultures. (C_1_) Representative traces of burst activity from WT cortical neuron cultures treated with DMSO, Tg, CHX, or CHX+Tg for 1 h at DIV14. Traces are from the same designated electrode ‘before’ and ‘after’ drug treatments. (C_2_) Quantification of burst duration by comparing ‘after treatment’ to ‘before treatment’ during the 15-min recordings from the same cultures. A two-way ANOVA with Tukey test was used. Data are represented as mean ± SEM with *P<0.05, ns: non-significant (n = 10–13 independent cultures).

To study the mechanisms by which ER stress reduces seizure severity, we asked whether the acute response to ER stress is able to modulate neural activity. To this end, we employed a MEA recording system to record extracellular spontaneous spikes of electrical activity in primary cortical neuron cultures prepared from WT mice. To determine the most appropriate time point for recordings, we measured the spontaneous spike frequency in cultures starting from days-in-vitro (DIV) 10 until DIV 18. As shown (S4 Fig), the spontaneous spike frequency peaks at DIV 14 and gradually goes down after DIV 16, which is consistently observed by another study [23], with no obvious changes in the number of active electrodes after DIV 14. These results suggest the network is likely most active and stable at DIV 14 in our cultures, as we have observed previously [18]. We therefore chose DIV 14, similar to other previous studies [24–27], to perform our recordings. To induce ER stress, we treated the cultures at DIV 14 with DMSO or Tg (1 μM) for one hour. This dosage and treatment duration were chosen based on previous studies showing effective induction of ER stress without triggering cell death [28–30]. The treatment duration was selected also because of our intent to focus on the acute response after the onset of ER stress. As shown in Fig 2, we observed a reduction in frequency, but not in amplitude, of spontaneous spikes in cultures treated with Tg in comparison to cultures treated with DMSO. When analyzing the firing pattern of spontaneous spikes, we observed no significant difference in burst activity (as demonstrated by the duration of qualifying bursts). The numbers of active electrodes were not changed after DMSO or Tg treatments (S5 Fig). These results suggest reduced spontaneous spikes in cortical neuron cultures during the early phase of ER stress.

Previous studies have linked multiple translational regulators to epileptogenesis [31] and neural activity regulation [32]. Because we confirmed that seizures can induce ER stress (Fig 1A) and it has been shown previously that a subset of proteins can be selectively translated during acute ER stress response [33], we asked whether the ER stress-induced reduction of spontaneous spikes are dependent on protein translation. As shown in Fig 2, pre-treatment with cycloheximide (60 μM)[34], a translation inhibitor, blocked the reduction of spontaneous spike frequency, without changes to spike amplitude or burst activity, upon the induction ER stress. The numbers of active electrodes were again not changed after drug treatments (S5 Fig). Altogether, our results indicate that ER stress, through a protein translation-dependent mechanism, reduces spontaneous spike frequency, which suggest a role for the ER stress response in reducing neural activity.

### Acute ER stress elevates the expression of Mdm2

**Fig 3:**
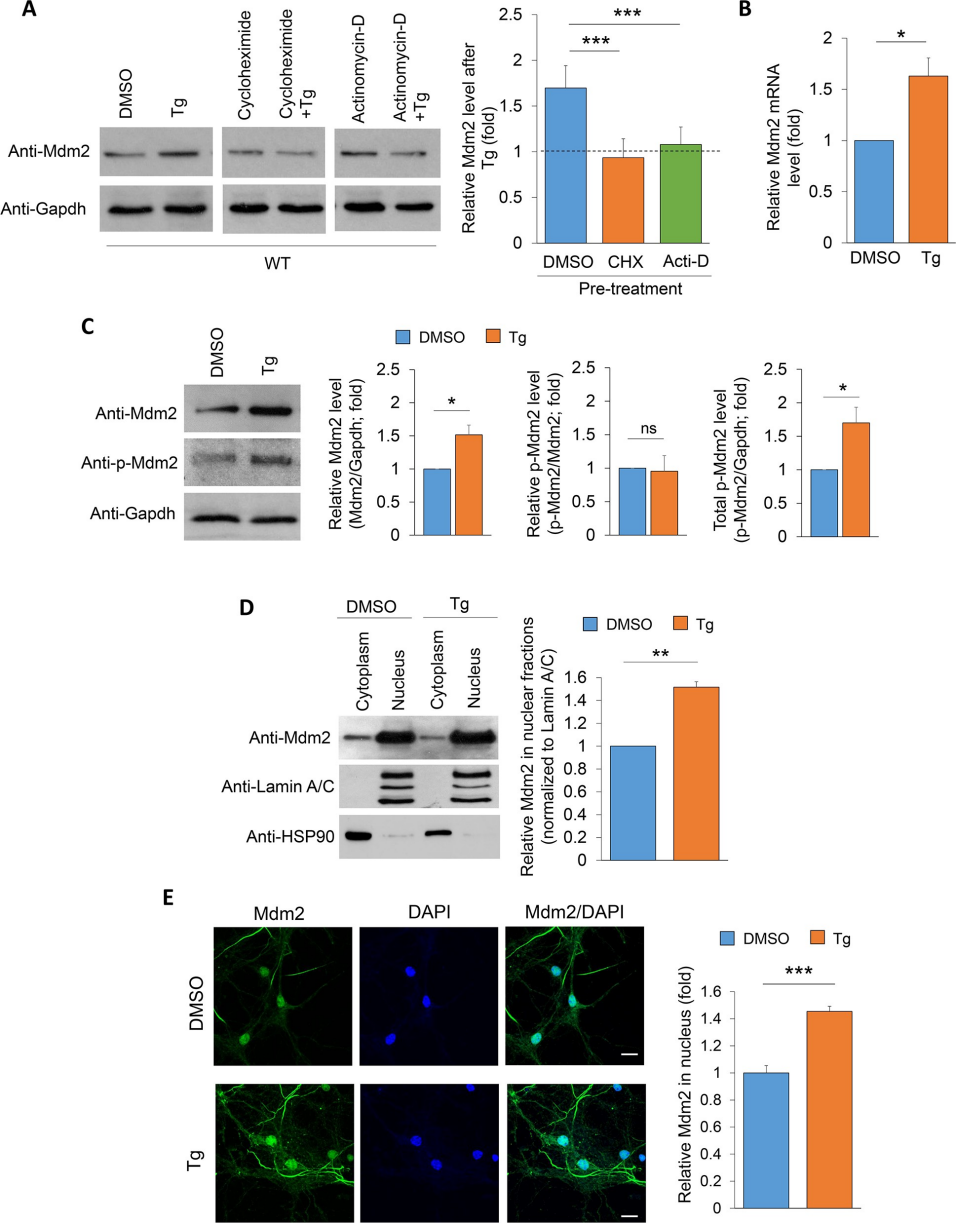
Mdm2 expression and nuclear accumulation are induced during the early phase of ER stress. (A) Representative western blots of Mdm2 and Gapdh after treatments of vehicle (DMSO) or Thapsigargin (Tg, 1 μM) for 1 hour in WT cortical neuron cultures pre-treated with DMSO, cycloheximide (60 μM) or actinomycin (20 μM) for 10 minutes. Quantification of Mdm2 level is on the right (n = 6). (B) Quantitative real-time RT-PCR of Mdm2 mRNA normalized to Actin mRNA from WT cortical neuron cultures treated with vehicle (DMSO) or Tg for 1 hour (n = 4). (C) Representative western blots of phospho (P)- Mdm2, Mdm2, and Gapdh from WT cortical neuron cultures treated with vehicle (DMSO) or Thapsigargin (Tg, 1 μM) for 1 hour (n = 7). (D) Representative western blots of Mdm2, Lamin A/C and HSP-90 after nuclear and cytosolic extraction using WT cortical neuron cultures treated with vehicle (DMSO) or Tg for 1 hour. Lamin A/C and HSP-90 serve as controls for nuclear and cytosolic fractions, respectively. Quantification is done after normalizing Mdm2 to Lamin A/C (n = 5). (E) Immunocytochemistry of WT cortical neuron cultures treated with vehicle (DMSO) or Thapsigargin (Tg, 1 μM) for 1 hour. Representative Mdm2, DAPI and merged images, as well as quantification of nuclear Mdm2, are shown (n = 20 cells from two independent cultures). Scale bar: 20 μm. For the quantification above, a one-way ANOVA with Tukey test (A) and Student’s *t*-test (B-E) were used. Data are represented as mean ± SEM with *P<0.05, **P<0.01, ***P<0.001, ns: non-significant.

To determine whether and how acute ER stress modulates protein translation, we studied murine double minute-2 (Mdm2), a ubiquitin E3 ligase known to participate in various cellular stress responses [35, 36]. As shown in Fig 3A, cortical neuron cultures treated with Tg (1 μM) for one hour show significantly elevated Mdm2 protein levels. However, this elevation was blunted by pre-treatment with either the translational inhibitor cycloheximide (60 μM) or a transcription inhibitor actinomycin-D (20 μM), indicating that the elevation of Mdm2 upon induction of ER stress likely occurs at the transcription level. To test this possibility, we performed real-time reverse transcription and quantitative PCR (real-time RT qPCR) to measure the relative levels of Mdm2 mRNA upon induction of ER stress. As shown in Fig 3B, cultures treated with Tg exhibit significantly elevated levels of Mdm2 mRNA. Together, our results indicate that induction of ER stress elevates Mdm2 expression.

The activity, substrate recognition and subcellular distribution of Mdm2 are known to be regulated by phosphorylation at serine-163 (serine-166 in human Mdm2) [37, 38]. To determine whether ER stress elevates the levels of phosphorylated Mdm2, we measured the phosphorylation of Mdm2 at serine-163 in WT cortical neuron cultures and found it was significantly elevated during ER stress; however, the relative level of phosphorylated Mdm2 (p-Mdm2/t-Mdm2) was unchanged (Fig 3C). These data suggest that the signals leading to Mdm2 phosphorylation are likely unchanged. Instead, it is that phosphorylated Mdm2 is proportionally increased with the up-regulation of total Mdm2, thus increasing the availability of phosphorylated Mdm2 during the early phase of the ER stress response.

Because Mdm2 phosphorylation at serine-163 leads to its nuclear distribution [37], we asked whether Mdm2 accumulates in the nucleus upon ER stress. As shown in Fig 3D, we found significant elevation of Mdm2 in the nuclear fraction upon ER stress induction for one hour in WT cortical neuron cultures. This observation was confirmed with immunocytochemistry where acute ER stress significantly elevated Mdm2 in the nucleus, which was identified by the staining of 4′,6-diamidino-2-phenylindole (DAPI) (Fig 3E). Altogether, our data show that acute ER stress elevates expression and nuclear accumulation of Mdm2.

### Acute ER stress promotes Mdm2-dependent p53 down-regulation and protein translation

**Fig 4:**
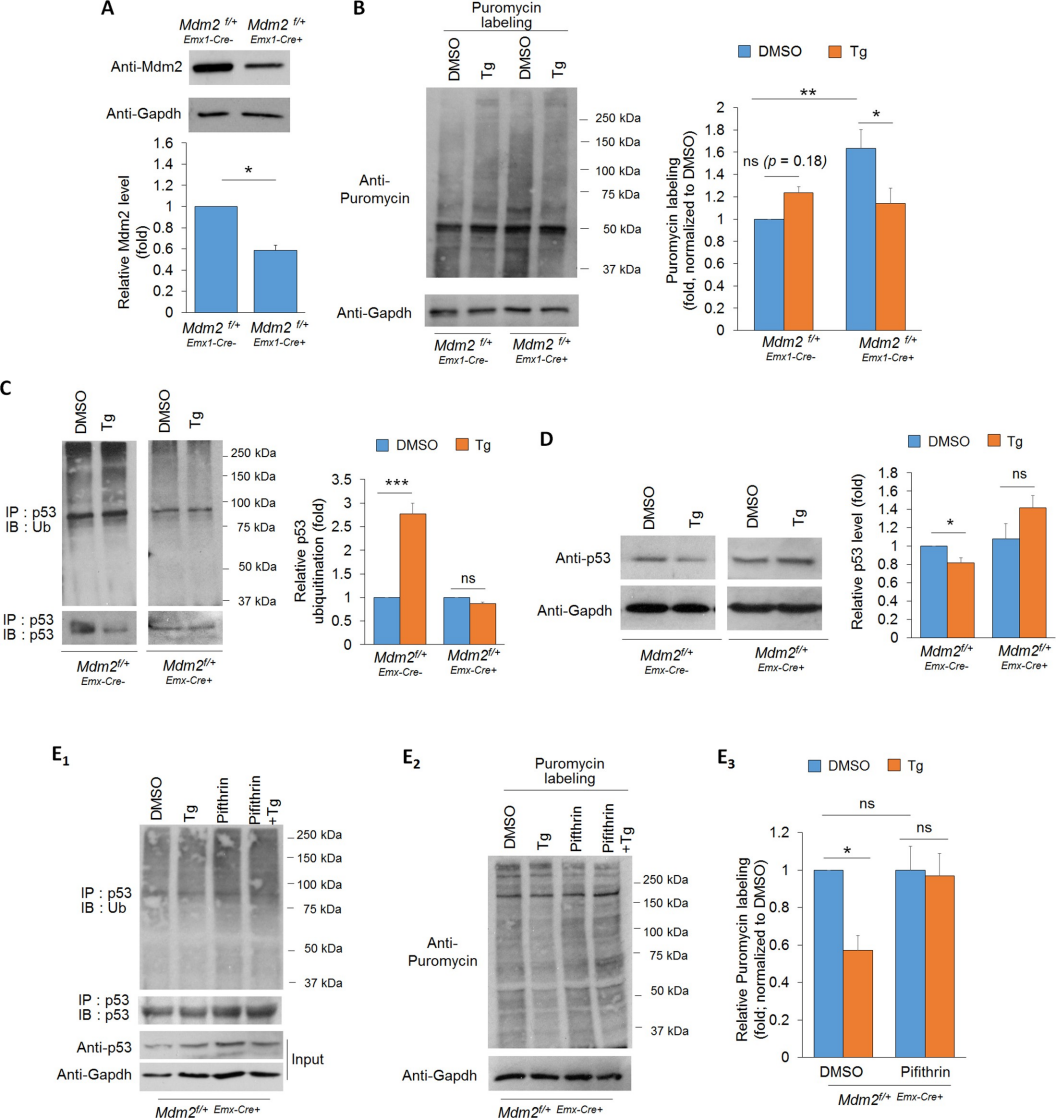
Mdm2 triggers ubiquitination and down-regulation of p53, and p53-dependent protein translation during the early phase of ER stress. (A) Quantification of Mdm2 expression and representative western blots of Mdm2 and Gapdh from *Mdm2*^*f/+*-*Emx1*-Cre-^ or *Mdm2*^*f/+*-*Emx1*-Cre+^ cortical neuron cultures at DIV14 (n = 4). (B) Representative western blots of puromycin and Gapdh, and quantification after 1-hour of puromycin labeling in *Mdm2*^*f/+*-*Emx1*-Cre-^ or *Mdm2*^*f/+*-*Emx1*-Cre+^ cortical neuron cultures treated with vehicle (DMSO) or Tg for 1 hour (n = 10). (C) Representative western blots of Ubiquitin and p53 after immunoprecipitation with anti-p53 antibody using lysates from *Mdm2*^*f/+*-*Emx1*-Cre-^ and *Mdm2*^*f/+*-*Emx1*-Cre+^ cortical neuron cultures treated with vehicle (DMSO) or Tg for 1 hour. Quantification is performed by first normalizing ubiquitinated p53 (IP:p53, IB:Ub) to immunoprecipitated p53 (IP:p53, IB:p53), followed by normalizing Tg-treated group to DMSO-treated group within the same genotype (n = 4). (D) Representative western blots of p53 and Gapdh from *Mdm2*^*f/+*-*Emx1*-Cre-^ or *Mdm2*^*f/+*-*Emx1*-Cre+^ cortical neuron cultures treated with vehicle (DMSO) or Tg for 1 hour. Quantification is shown on the right (n = 7). (E) Representative western blots of Ubiquitin and p53 after immunoprecipitation with anti-p53 antibody in *Mdm2*^*f/+*-*Emx1*-Cre+^ cortical neuron cultures pre-treated with vehicle (DMSO) or Pifithrin-α (1 μM) for 10 minutes, followed by treatment with vehicle (DMSO) or Thapsigargin (Tg, 1 μM) for one hour (E_1_); and representative western blots of puromycin and Gapdh after 1-hour labeling of puromycin in *Mdm2*^*f/+*-*Emx1*-Cre+^ cortical neuron cultures pre-treated with vehicle (DMSO) or Pifithrin-α (1 μM) for 10 minutes, followed by treatment with vehicle (DMSO) or Thapsigargin (Tg, 1 μM) for one hour (E_2_). Quantification of puromycin labeling is shown on the right (n = 6). For the quantification, Student’s *t*-test (A) and a two-way ANOVA with Tukey test (B-E) were used. Data are represented as mean± SEM with *P<0.05, **P<0.01, ***P<0.001, ns: non-significant.

To examine whether Mdm2 modulates protein translation during ER stress, we employed a conditional Mdm2 knockdown mouse model by crossing Mdm2 floxed mice (*Mdm2*^f/f^) with *Emx1*-Cre mice to obtain *Mdm2*^*f/+-Emx-Cre+*^ and *Mdm2*^*f/+-Emx-Cre-*^ mice. *Emx1*-Cre can confer Mdm2 reduction in the cortex and hippocampus, primarily in excitatory neurons, beginning as early as embryonic day 10.5 (E10.5) [39, 40]. We used heterozygous mice (*Mdm2*^*f/+*^) to avoid potential apoptosis caused by complete Mdm2 knockout [41, 42]. The knockdown efficiency of Mdm2 in *Mdm2*^*f/+-Emx-Cre+*^ cortical neuron cultures at DIV 14 is approximately 42% in comparison to the *Mdm2*^*f/+*-*Emx1*-Cre-^ cultures, when cultures are prepared on postnatal day 0 (Fig 4A). We then determined global protein translation in *Mdm2*^*f/+*-*Emx1*-Cre+^ and *Mdm2*^*f/+*-*Emx1*-Cre-^ cultures after the induction of ER stress for one hour, with the use of puromycin to label newly synthesized proteins followed by western blotting with an anti-puromycin antibody. As shown in Fig 4B, induction of ER stress in *Mdm2*^*f/+*-*Emx1*-Cre-^ cultures slightly elevated global protein translation. Because the typical cellular response to ER stress is traditionally known to repress protein translation, we suspect that such elevation, or maintenance, of translation only occurs during the early phase (1 hour) of ER stress. To test this hypothesis, we treated the cortical neuron cultures with DMSO or Tg for 4 hours, a duration of treatment known to repress protein translation [43, 44]. As shown in S6 Fig, the cultures that received Tg for 4 hours, with puromycin labeling occurring only during the fourth hour, exhibit a reduction in global protein translation. These results confirm an elevation, or maintenance, of protein translation during the early phase of the ER stress response in primary cortical neuron cultures.

In contrast to *Mdm2*^*f/+*-*Emx1*-Cre-^ cultures, we observed basally elevated translation in *Mdm2*^*f/+*-*Emx1*-Cre+^ cultures (Fig 4B), consistent with our previous study showing that Mdm2 acts as a translational suppressor [34]. Of note, the acute ER stress-induced maintenance of protein translation was not observed in *Mdm2*^*f/+*-*Emx1*-Cre+^ cultures (Fig 4B). Instead, a significant down-regulation of protein translation was observed. Altogether, our data suggest that Mdm2 is required to promote or maintain protein translation during the early phase of ER stress in cortical neuron cultures.

Elevated Mdm2 upon ER stress was initially speculated to reduce protein translation, since Mdm2 has previously been found to interact with ribosomes and repress translation [34]. Because we instead observed an elevation of protein translation upon ER stress, we suspected that Mdm2 regulates translation through a different mechanism. Because we observed nuclear accumulation of Mdm2 upon ER stress and because Mdm2 is known to ubiquitinate its substrate tumor suppressor p53 in the nucleus [45, 46], we asked whether induction of ER stress leads to ubiquitination and down-regulation of p53 through Mdm2. As shown in Fig 4C, an elevation of p53 ubiquitination was observed in *Mdm2*^*f/+*-*Emx1*-Cre-^ cultures after Tg treatment for one hour. In *Mdm2*^*f/+*-*Emx1*-Cre+^ cultures, we instead saw a slight reduction of p53 ubiquitination. These results of p53 ubiquitination were consistent with the total protein level of p53, where a significant reduction of p53 was seen in *Mdm2*^*f/+*-*Emx1*-Cre-^ cultures but a slight trend toward elevation of p53 was seen in *Mdm2*^*f/+*-*Emx1*-Cre+^ cultures after Tg treatments (Fig 4D). These results confirmed Mdm2-dependent p53 down-regulation during the early phase of the ER stress response. In contrast, elevated expression of Mdm2 and the subsequent down-regulation of p53 were not observed in cultures treated with Tg for 4 hours (S7 Fig) despite the continuous ER stress response shown by elevated phosphorylation of eIF2α (S8 Fig). These results suggest that Mdm2-triggered ubiquitination and down-regulation of p53 only occur during the early phase of the ER stress response.

Because our observation of Mdm2-dependent p53 down-regulation (Fig 4C and 4D) is consistent with our data on protein translation during early phase of the ER stress response (Fig 4B), we suspect that impaired down-regulation of p53 is responsible for the failure to maintain protein translation during the early phase of ER stress in *Mdm2*^*f/+*-*Emx1*-Cre+^. To test this possibility, we employed a widely used p53 transcriptional inhibitor Pifithrin-α (1 μM) [45, 47–49] in *Mdm2*^*f/+*-*Emx1*-Cre+^ cultures to mimic inactivation of p53 when degraded in *Mdm2*^*f/+*-*Emx1*-Cre-^ cultures (Fig 4D). As shown in Fig 4E, *Mdm2*^*f/+*-*Emx1*-Cre+^ cultures pre-treated with Pifithrin-α were able to maintain protein translation upon induction of ER stress, as seen in *Mdm2*^*f/+*-*Emx1*-Cre-^cultures (Fig 4B), supporting our conclusion that ER stress-induced down-regulation of p53 allows for maintenance of protein translation.

It is unclear how stabilized p53 can disrupt protein translation during ER stress in *Mdm2*^*f/+*-*Emx1*-Cre+^ cultures. One possibility is through reduction of ribosome biogenesis as previously described [50]. To test this possibility, we characterized ribosome biogenesis through the measurement of 47S pre-rRNA, as shown previously [51, 52], of *Mdm2*^*f/+*-*Emx1*-Cre-^ and *Mdm2*^*f/+*-*Emx1*-Cre+^ cortical neuron cultures after the induction of ER stress for one hour. However, as shown in S9 Fig, we did not detect significant changes in the levels of 47S pre-rRNA that could explain the protein translation phenotypes observed in *Mdm2*^*f/+*-*Emx1*-Cre+^ cultures. These data suggest that Mdm2-p53 signaling likely acts through a novel, and yet unidentified, mechanism to maintain protein translation during the early phase of ER stress response.

### Inhibition of p53 restores reduction of neural activity induced by acute ER stress in Mdm2 conditional knockdown neurons

**Fig 5:**
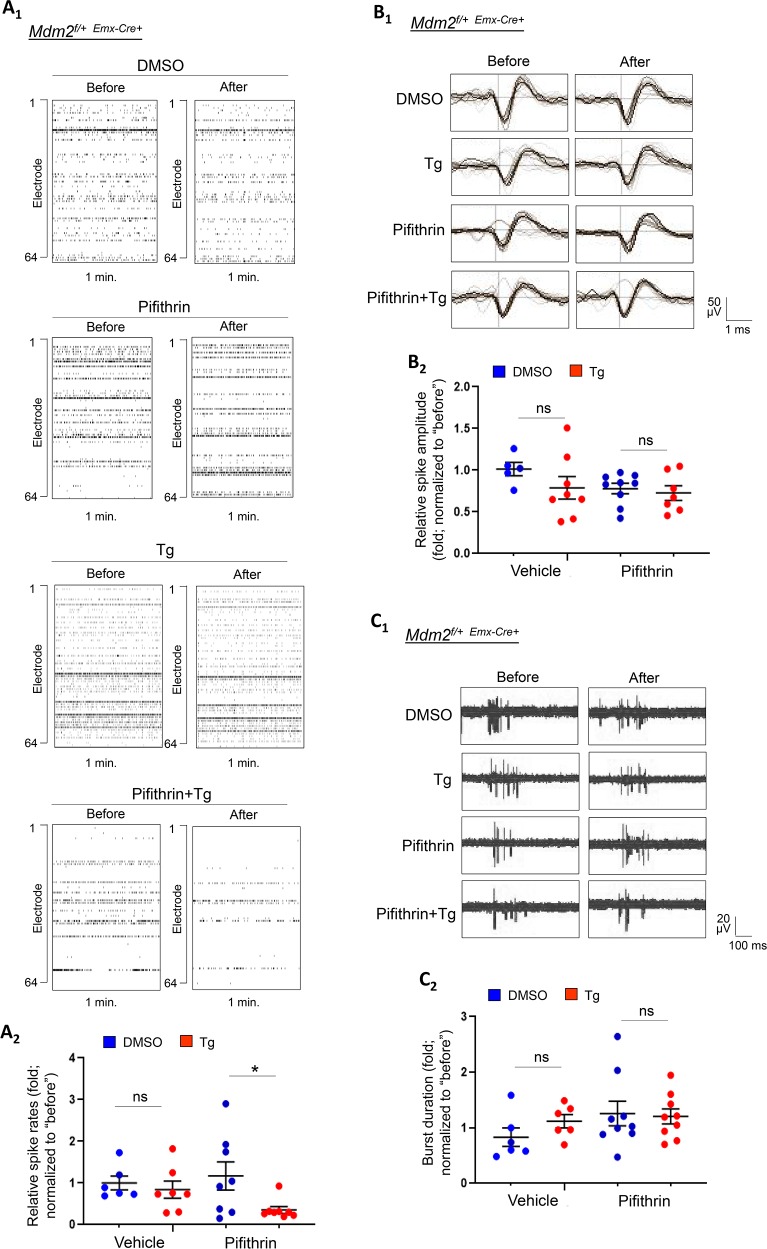
Inhibition of p53 restores aberrant neural network activity during the early phase of ER stress in *Mdm2*^*f/+-Emx1*-Cre+^ cortical neuron cultures. (A_1_) Raster plots of spontaneous spikes from representative 1-min recordings of *Mdm2*^*f/+*-*Emx1*-Cre+^ cortical neuron cultures pre-treated with vehicle (DMSO) or Pifithrin-α (1 μM) for 10 minutes, followed by treatment with vehicle (DMSO) or Thapsigargin (Tg, 1 μM) for 1 hour at DIV14. (A_2_) Quantification of relative spontaneous spike rates by comparing ‘after treatment’ to ‘before treatment’ of the same cultures during the 15-min recordings. (B_1_) Representative average traces of spike amplitude from 1-min recordings of *Mdm2*^*f/+*-*Emx1*-Cre+^ cortical neuron cultures pre-treated with DMSO or Pifithrin-α for 10 minutes, followed by treatment with DMSO or Tg for 1 hour at DIV14. In the traces, the black lines represent the average of all the spikes within representative 1-min recordings. Traces are from the same designated electrodes before and after treatments. (B_2_) Quantification of average spontaneous spike amplitude by comparing ‘after treatment’ to ‘before treatment’ during the 15-min recordings of the same cultures. (C_1_) Representative traces of burst activity from *Mdm2*^*f/+*-*Emx1*-Cre+^ cortical neuron cultures pre-treated with DMSO or Pifithrin-α for 10 minutes, followed by treatment with DMSO or Tg for 1 hour at DIV14. Traces are from the same designated electrode ‘before’ and ‘after’ drug treatments. (C_2_) Quantification of burst duration by comparing ‘after treatment’ to ‘before treatment’ during the 15-min recordings from the same cultures (n = 5–9 independent cultures). A two-way ANOVA with Tukey test was used. Data are represented as mean ± SEM with * P<0.05, ns: non-significant.

Our previous data suggest that the neuronal response to acute ER stress leads to a reduction in spontaneous spike frequency through a protein translation-dependent mechanism (Fig 2), and we have now shown that ER stress induces Mdm2-p53 signaling-dependent protein translation (Figs 3 and 4). To validate the role of Mdm2-p53 signaling in reduced neural activity triggered by acute ER stress, we performed MEA recording using *Mdm2*^*f/+*-*Emx1*-Cre+^ cultures. As shown in Fig 5A, Tg-induced reduction of spontaneous spike frequency was absent in *Mdm2*^*f/+*-*Emx1*-Cre+^ cultures, similar to WT cultures treated with the translation inhibitor cycloheximide (Fig 2). Spontaneous spike amplitude and burst activity were not affected (Fig 5B and 5C). These observations suggest that *Mdm2*^*f/+*-*Emx1*-Cre+^ cultures have lost the ability to reduce neural activity upon ER stress induction. However, most importantly, the deficits of *Mdm2*^*f/+*-*Emx1*-Cre+^ cultures in spontaneous spike frequency can be restored by pre-treatment with p53 inhibitor Pifithrin-α, thereby confirming the role of Mdm2-p53 signaling in these effects. The spontaneous spike amplitude and burst activity were not affected by Pifithrin-α (Fig 5B and 5C). To validate that our observation with the use of Pifithrin-α is specific to p53 and to provide a secondary method to confirm the role of p53 in the ER stress-induced reduction of neural activity, we lentivirally transduced a shRNA against p53 or a control non-target shRNA into *Mdm2*^*f/+*-*Emx1*-Cre+^ cultures. As shown (S10 Fig), knocking down approximately 40% of p53 was able to restore the Tg-induced reduction of spontaneous spike frequency in *Mdm2*^*f/+*-*Emx1*-Cre+^ cultures, again without changes in spontaneous spike amplitude or burst activity. Altogether, our results confirm the role of the Mdm2-mediated p53 down-regulation in ER stress-induced maintenance of protein translation and reduction of neural activity.

### Inhibition of p53 restores ER stress-induced reduction of seizure severity in Mdm2 conditional knockdown mice

**Fig 6:**
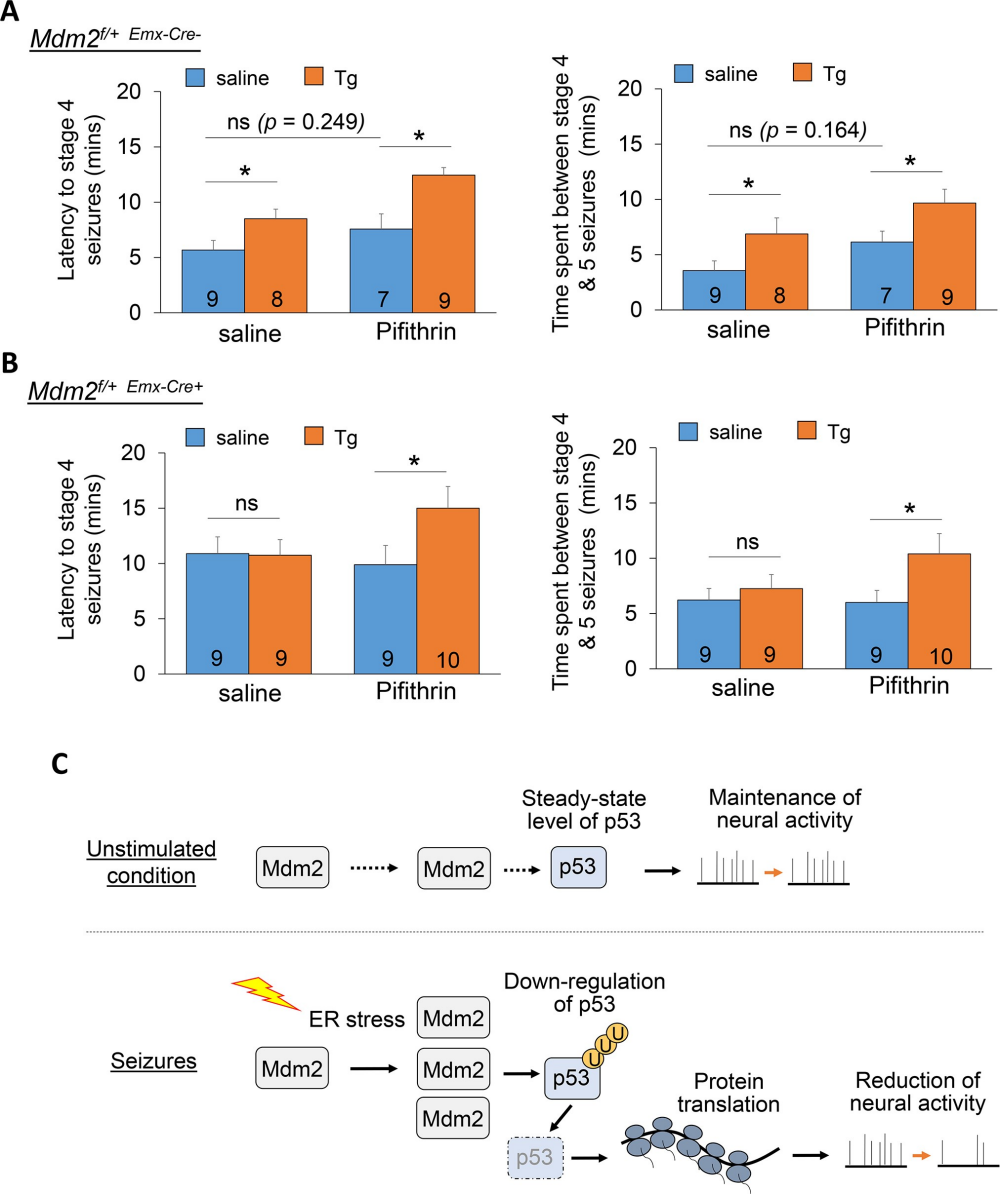
Inhibition of p53 restores the sensitivity of seizure severity to acute ER stress in *Mdm2*^*f/+-Emx1*-Cre+^ mice. (A, B) Quantification of latency to stage 4 seizures, and the time spent between stage 4 to 5 seizures from 3-week old *Mdm2*^*f/+*-*Emx1*-Cre-^ (A) or *Mdm2*^*f/+*-*Emx1*-Cre+^ (B) mice intraperitoneally injected with saline or Thapsigargin (Tg, 2 mg/kg) for 3 hours followed by injections with KA (60 mg/kg). The number of mice used in each condition is shown on the bottom of each bar. A two-way ANOVA with Tukey test was used. Data are represented as mean ± SEM with *P<0.05, ns: non-significant. (C) A working model showing Mdm2-p53 signaling under an unstimulated condition (top) and during seizure-induced ER stress (bottom), which triggers Mdm2-dependent ubiquitination and downregulation of p53, leading to protein translation-dependent reduction of neural activity.

Our data show that inducing acute ER stress prior to kainic acid stimulation can reduce seizure severity in mice (Fig 1). Based on our data which demonstrate the necessity of the Mdm2-p53 signaling pathway in the ER stress-induced reduction of neural activity (Fig 5), we next sought to determine whether the acute ER stress-induced reduction of seizure severity is altered in *Mdm2*^*f/+*-*Emx1*-Cre+^ mice and whether any deficits can be corrected by Pifithrin-α. We first confirmed an approximately 20% reduction in Mdm2 in the cortex of *Mdm2*^*f/+*-*Emx1*-Cre+^ mice when compared to that in *Mdm2*^*f/+*-*Emx1*-Cre-^ mice (S11 Fig). This moderate knockdown efficiency is likely due to the restrictive expression of *Emx1*-Cre to excitatory neurons while Mdm2 is also expressed in other cell types [53, 54]. Next, we intraperitoneally injected *Mdm2*^*f/+*-*Emx1*-Cre-^ or *Mdm2*^*f/+*-*Emx1*-Cre+^ mice with saline, Tg (2 mg/kg), Pifithrin-α (2 mg/kg), or Tg + Pifithrin-α. Three hours after initial injection, the mice were injected with kainic acid (60 mg/kg). Immediately following, seizure behavior was closely monitored and quantified. As shown in Fig 6A and 6B, the *Mdm2*^*f/+*-*Emx1*-Cre-^ mice who received Tg exhibited significantly delayed onset of stage 4 seizures and an extended time between stage 4 and stage 5 seizures, as we had observed in WT mice (Fig 1). Pifithrin-α slightly delayed the seizure activity, as we observed previously [17]. As we expected, the *Mdm2*^*f/+*-*Emx1*-Cre+^ mice who received Tg did not exhibit the delayed onset of stage 4 seizures or elevated duration between stage 4 and 5 seizures that were observed in *Mdm2*^*f/+*-*Emx1*-Cre-^ mice. Importantly, *Mdm2*^*f/+*-*Emx1*-Cre+^ mice receiving Tg + Pifithrin-α exhibited delayed onset of stage 4 seizures and enhanced duration between stage 4 and 5 seizures, supporting our previous observation that inhibition of p53 restores Tg-induced reduction of neural activity in *Mdm2*^*f/+*-*Emx1*-Cre+^ cortical neuron cultures (Fig 5 and S10 Fig). Basally elevated seizure latency was also observed in *Mdm2*^*f/+*-*Emx1*-Cre+^ mice in comparison to *Mdm2*^*f/+*-*Emx1*-Cre-^ mice, suggesting a possibility that Mdm2-p53 signaling pathway may regulate brain excitability through certain p53 target genes, such as potassium channel KCNKs [55], or through other unidentified mechanisms. Altogether, our data demonstrate that ER stress triggers Mdm2-p53 signaling-dependent protein translation which functions to reduce neural activity in cultures and seizure severity in mice (Fig 6C).

## Discussion

Our study revealed that acute responses to ER stress serve to reduce neural activity through a protein translation-dependent mechanism. Because we (Fig 1) and others [14] have shown that seizures can induce ER stress, our findings suggest that acute ER stress can initiate cellular responses that possess a beneficial function to counteract seizure activity. The data further support the notion that the cellular response to ER stress enables the neuron to deal with insults rather than passively waiting for the removal of the insults. A similar beneficial function has been reported in the past through the Mdm2-p53 signaling-dependent up-regulation of CCAAT/enhancer-binding protein homologous protein (CHOP) in the brain [3]. This study demonstrated an elevation of CHOP in the hippocampus of epileptic mice, and showed that knocking down CHOP expression was able to promote seizure-induced neuronal death and cognitive decline [3]. Both this previous study and our current work caution against inhibition of ER stress or Mdm2-p53 signaling as a neuroprotective strategy, especially in pathological conditions where excessive neural activity is observed. Both the positive (neural activity homeostasis) and negative (neuronal death) consequences of ER stress would need to be considered when developing therapies against hyperexcitability or excitotoxicity.

Because our results show a beneficial effect of ER stress response toward reducing seizure severity after a chronic induction of ER stress, it would be of particular interest to determine how a disruption or promotion of ER stress may affect the duration or frequency of subsequent seizures in individuals with reoccurring seizures. To address this important issue, it would be useful to measure long-term seizure activity in a model of reoccurring seizures with an electrocephalogram (EEG), which provides more detailed and sensitive measurements of seizure activity than behavioral studies due to the unpredictable and often behaviorally unnoticeable nature of spontaneous reoccurring seizures. Related to this question, it remains unknown whether the intensity of ER stress and the beneficial function of Mdm2-p53 signaling remain the same each time a seizure occurs. It is assumed that ER stress and Mdm2-p53 signaling can be induced during each seizure. However, it is unknown whether there are any compensatory mechanisms or whether desensitization occurs after repeated seizures. In addition, because the duration and severity of seizures are extremely variable in patients, how long ER stress lasts after seizures and how long it takes for the neuron to recover from seizure-induced ER stress are likely variable, as well. Presumably, based on our results, promoting ER stress could help reduce excitability in the brain. However, given the complexity of human epilepsies, our limited knowledge about ER stress in the nervous system, as well as the multitude of functions of Mdm2-p53 signaling in the neuronal cells, manipulating ER stress to reduce brain excitability may not be ideal at this moment. The full effect of ER stress and a detailed time course of the ER stress response in neurons needs to be further explored in order to better understand excitability homeostasis, especially in epilepsy patients.

Our study showed that ER stress-mediated neural activity reduction depends on protein translation. ER stress is traditionally known to activate pathways which repress translation, such as phosphorylation of eIF2α, with the rationale that doing so conserves energy consumption [56]. Our results demonstrated a maintenance in protein translation mediated by Mdm2-p53 signaling during the early phase of the ER stress response, suggesting the possibility that Mdm2-p53 signaling offsets the translational suppression mediated by eIF2α phosphorylation or other pathways. When Mdm2-p53 signaling is turned off during the late phase of the ER stress response, translational suppression dominates and is then apparent. If this prediction is true, it would suggest Mdm2-p53 signaling is an important “switch” for turning protein translation on and off during different phases of ER stress. Studies have also indicated that certain proteins, particularly those associated with cellular stress response such as heat shock proteins, continue to be translated during ER stress [33]. Based on this rationale, it is likely that certain proteins associated with the reduction of neuronal or synaptic excitability could be preferentially translated during the early phase of ER stress in order to reduce neural activity. These proteins could include ion channels, neurotransmitter receptors, and many others. A comprehensive proteomic profiling is the logical next step to identify the proteins being translated within neurons during the early phase of ER stress. Alternatively, the use of slice electrophysiology, which allows for wash-out experiments with drugs, could also help to dissect the relative contribution of synaptic transmission or intrinsic excitability to ER stress-induced reduction of neural activity, as well as the associated ionic mechanisms. Such analyses will provide novel insights into the cellular stress response in terminally differentiated neuronal cells.

Our current results indicate a novel, although indirect, mechanism by which Mdm2 regulates translation: through ubiquitination and down-regulation of its nuclear substrate p53 to maintain translation. Our data have ruled out ribosome biogenesis as the mechanism underlying p53-dependent protein translation during ER stress, but the precise mechanism remains unidentified. A previously published unbiased genome-wide study has identified several target genes of p53 that could potentially modulate protein translation through the ribosomal protein S6 kinase (S6K) pathway [55], including *Notch1*, *TGF-α* and *FGF-2* [57–59]. A prominent future direction would be studying the expression and regulation of these genes, and their roles in modulating S6K activity during the ER stress response.

Our previous study identified Mdm2 as a translational suppressor that acts through directly binding to ribosomes in the cytoplasm [34]. It is logical to speculate that if ER stress becomes unresolved after a long period of time and translational suppression is required, Mdm2 might re-distribute from the nucleus to the cytoplasm to increase Mdm2-ribosome interaction. This would also allow for stabilization of p53 in the nucleus, where it could suppress cellular growth and be ready to elicit apoptosis when needed. This prediction is supported by our data showing diminished elevation of total Mdm2 and nuclear Mdm2 after chronic induction of ER stress (S5 Fig). It is also supported by studies which have shown that the nuclear export of Mdm2 requires dephosphorylation by the protein phosphatase PP2A [37] and ER stress is known to activate PP2A and its downstream signaling pathways [60, 61]. Based on our current study showing the connection between Mdm2 and ER stress-dependent regulation of neural activity and our previous studies showing a role for Mdm2-dependent signaling in homeostatic plasticity [45, 46], we believe Mdm2 is a crucial molecule in brain excitability homeostasis and should be studied further. Given that many therapeutic agents targeting Mdm2 or Mdm2-p53 signaling are clinically available or being developed, a better understanding of Mdm2 would benefit future therapeutic development for seizures, epilepsies, and other neurological conditions associated with excessive neural activity.

## Materials and methods

### Ethics statement

All experiments using animal data followed the guidelines of Animal Care and Use provided by the Illinois Institutional Animal Care and Use Committee (IACUC) and the guidelines of the Euthanasia of Animals provided by the American Veterinary Medical Association (AVMA) to minimize animal suffering and the number of animals used. This study was performed under an approved IACUC animal protocol of University of Illinois at Urbana-Champaign (#17075 to N.-P. Tsai.)

### Animals

The WT (C57BL/6J) and *Emx1*-Cre mice were obtained from The Jackson Laboratory. The Mdm2-floxed mice were obtained from Frederick National Laboratory for Cancer Research.

### Reagents

Cycloheximide was from Santa Cruz Biotechnology. Actinomycin-D was from Sigma. MG132 was from Selleck Chemical. Puromycin was from MP Biomedicals. Kainic acid and Thapsigargin were from Alomone Lab. Pifithrin-α was from Adipogen Corporation. Salubrinal was from Enzo Life Sciences. Dimethyl sulfoxide (DMSO) was from Fisher Scientific. DMSO was used as a vehicle in this study. The antibodies used in this study were purchased from Santa Cruz Biotechnology (anti-Mdm2, anti-Lamin A/C, anti-HSP-90), GenScript Corporation (anti-Gapdh), Millipore (anti-puromycin), and Cell Signaling (anti-ubiquitin, anti-p-Mdm2, anti-p53, anti-BiP, anti-PDI, anti-PERK, anti-ATF6, anti-XBP1s, anti-eIF2α and anti-p-eIF2α). HRP-conjugated secondary antibodies were from Santa Cruz Biotechnology and Cell Signaling. The lentiviral control non-target shRNA and p53 shRNA were from Santa Cruz Biotechnology.

### Primary neuron cultures

Primary neuron cultures were made from mice aged at p0-p1 as described previously [34] and maintained in Neural Basal-A medium (Thermo Fisher) supplemented with B27 supplement (Thermo Fisher), GlutaMax (final concentration at 2 mM; Thermo Fisher), and Cytosine β-D-arabinofuranoside (AraC, final concentration at 2 μM; Sigma). AraC was added 6 hours after plating to prevent glial overgrowth. To prevent unwanted synchronous activity triggered by Penicillin [62–64], no antibiotics were added to our cultures. The culture medium was changed 50% on DIV 2 and every 3–4 days thereafter until the experiments on DIV 14.

### MEA recording

MEA recordings were performed as previously described [65]. In brief, each MEA plate was coated with poly-D-lysine for 30 minutes and plated with 2x10^5^ cells counted using a hemocytometer. Recordings were done at DIV 13–14 in the same culture medium using an Axion Muse 64-channel system in single well MEAs (M64-GL1-30Pt200, Axion Biosystems) inside a 5% CO_2_, 37°C incubator. Field potentials (voltage) at each electrode relative to the ground electrode were recorded with a sampling rate of 25 kHz. After 30 min of baseline recording, the MEA was treated with the drugs indicated in each experiment and recorded for another 30 min. Because of the changes in network activity caused by physical movement of the MEA when starting each recording, only the last 15 min of each recording were used in data analyses. AxIS software (Axion Biosystems) was used for the extraction of spikes from the raw electrical signal obtained from the Axion Muse system. After filtering, a threshold of ±7 standard deviations was independently set for each channel; activity exceeding this threshold was counted as a spike. Only MEAs with more than 2,000 spikes during the last 15 minutes of recording were included for data analysis [17, 66]. The total spikes obtained from each MEA culture was normalized to the number of electrodes, as described in a previous study [67]. The settings for burst detection in each electrode were a minimum of 5 spikes with a maximum inter-spike interval of 0.1 sec as described previously [17]. The burst duration and number of spikes per burs were analyzed by AxIS software.

To ensure consistency when acquiring MEA data, all experiment procedures, including the animal dissection, cell counting and plating, medium changing, and recordings are conducted by the same individual in each experiment. Throughout culture maturation and before recording, each MEA is visually inspected under the microscope and any MEA with poor growth is excluded. Recordings of each experiment were alternate between treatments or genotypes. For all before and after drug treatment comparisons, to minimize the variability between cultures, the recording from each MEA culture after treatment was compared to the baseline recording from that same culture.

### Seizure induction and seizure activity test

Male mice at age 3-weeks or 12-weeks old were intraperitoneally injected with kainic acid, prepared in saline solution (Hannas Pharmaceutical), at doses of 30 mg/kg or 60 mg/kg as indicated in each figure. The total injection volume was kept close to 0.1 ml. After injection, mice were closely observed in real time for 1 hour. The intensity of seizures was assessed by a modified Racine’s scoring system [68]. To clearly determine seizure activity, only stage 4 (rearing and falling) and stage 5 (tonic-clonic activity) were considered positive for seizures, as previously performed [17, 18, 69]. The mortality rate after seizures is summarized in S1 Table.

### Immunoprecipitation and western blotting

For immunoprecipitation (IP), cell lysates were obtained by sonicating pelleted cells in IP buffer (50 mM Tris, pH 7.4, 120 mM NaCl, 0.5% Nonidet P-40). Eighty μg of total protein mixtures were incubated for one hour at 4°C with 0.5 μg primary antibodies. Protein A/G agarose beads were added for another hour followed by washing with IP buffer three times. For western blotting, after SDS-PAGE, the gel was transferred onto a polyvinylidene fluoride membrane (Santa Cruz Biotechnology). After blocking with 1% Bovine Serum Albumin in TBST buffer (20 mM Tris pH 7.5, 150 mM NaCl, 0.1% Tween-20), the membrane was incubated with primary antibody overnight at 4°C, followed by three 10-min washings with TBST buffer. The membrane was then incubated with an HRP-conjugated secondary antibody for 1 hour at room temperature, followed by another three 10-min washings. Finally, the membrane was developed with an ECL Chemiluminescent Reagent [70]. All the western blot results were semi-quantitatively normalized to the control groups within the same set of sister cultures for analysis.

### Nuclear and cytoplasmic fractionation

Nuclear/cytoplasmic fractionation was conducted using the NE-PER Nuclear and Cytoplasmic Extraction Reagents Kit (Pierce Chemical) as described previously [37] and according to manufacturer’s protocol. Lamin A/C and HSP-90 served as nuclear and cytoplasmic markers, respectively.

### Real-time quantitative reverse transcription PCR (RT-qPCR)

After drug treatment, the total RNA from cortical neurons in cultures was obtained with TRIzol reagent (Life Technologies). Reverse transcription was performed with Photoscript reverse transcriptase (New England Biolab) and the real-time PCR was performed with Thermo Scientific Maxima SYBR Green reagent. The primers used in this study were: Mdm2, 5’- AGC AGC GAG TCC ACA GAG A -3’ and 5’- ATC CTG ATC CAG GCA ATC AC -3’; Actin, 5’- CCT GTG CTG CTC ACC GAG GC -3’ and 5’- GAC CCC GTC TCT CCG GAG TCC ATC -3’; 47S, 5’- CGT GTA AGA CAT TCC TAT CTC G-3’ and 5’- GCC CGC TGG CAG AAC GAG AAG-3’.

### Immunocytochemistry

Immunocytochemistry was done as previously described [18]. In brief, primary neurons grown on poly-D-lysine coated coverslips were fixed at DIV 14 with ice-cold buffer (4% paraformaldehyde and 5% sucrose in PBS). After washing and permeabilization with an additional incubation with 0.5% Triton X-100 in PBS for 5 min, an incubation with anti-Mdm2 antibody was performed overnight. After washing three times with PBS, fluorescence-conjugated secondary antibodies were applied to the cells at room temperature for 1 hour. After washing the cells an additional three times with PBS, the coverslips were mounted using a mounting medium (Fisher Scientific) supplied with DAPI and observed under Zeiss LSM 700 Confocal Microscope with 40X magnification. Pinhole was set to 1 airy unit for all experiments. Confocal microscope settings were kept with the same laser and scanning configurations to allow for comparison across conditions. Quantification was done by first circling the nuclei using the line tools in ImageJ (National Institute of Health) based on the DAPI signal. Subsequently, the Mdm2 signal in circled regions was measured also using ImageJ software before subtracting background signal. The final Mdm2 signal from the Tg-treated group is then normalized to that from the vehicle-treated group.

### Statistical analysis

All the numerical data underlying graphs were summarized in S1 Appendix. The data presented in this study have been tested for normality using Kolmogorov-Smirnov Test. Statistical methods to determine significance along with sample numbers were indicated in each figure legend. In brief, ANOVA with post-hoc Tukey HSD (Honest Significant Differences) test was used for multiple comparisons between treatments or genotypes (Figs 2, 3A, 4B–4E, 5, 6A and 6B, S1 Fig and S5 Fig). Student’s *t*-test was used for the conditions where only two treatment groups were performed (Figs 1, 3B–3D, 4A, S2 Fig, S3 Fig, S6 Fig, S7 Fig, S8 Fig, S9 Fig, S10 Fig and S11 Fig.). Each “n” indicates an independent culture. Differences are considered significant at the level of *p* < 0.05.

## Supporting information

S1 Fig Confirmation of ER stress after injections of Thapsigargin or Salubrinal in mice. Quantification and representative western blots of BiP, PDI, PERK, XBP1s, ATF6 and Gapdh from total brain lysate of 3-week old WT mice intraperitoneally injected with saline, Thapsigargin (Tg, 2 mg/kg) or Salubrinal (Sal, 2 mg/kg) followed by kainic aicd (60 mg/kg). The arrow indicates the predicted position for cleaved ATF6. A one-way ANOVA with Tukey test was used. Data are represented as mean ± SEM with **P<0.01, ***P<0.001, ns: non-significant (n = 4). Of note, despite an elevation of XBP1s in Tg-treated groups, the mice treated with Salubrinal did not show reduction of XBP1s when compared to the mice treated with saline only. This is likely due to the fact that Salubrinal functions to inhibit eIF2α pathway and may not affect XBP1 splicing.
(TIF)

S2 Fig Induction of ER stress reduces seizure severity in adult mice. Quantification of latency to stage 4 seizures, and the time spent between stage 4 to 5 seizures from 12-weeks old WT mice intraperitoneally injected with saline or Thapsigargin (Tg, 2 mg/kg) for 3 hours followed by injections with KA (60 mg/kg). Injections of kainic acid with 30 mg/kg were not included in this experiment because preliminary tests showed no signs of stage 5 seizures in adult WT mice, likely because the resistance to KA-induced seizures is more apparent in adult mice of C57BL/6J background. The number of mice used in each condition is shown on the bottom of each bar. For the quantification, Student’s *t*-test was used. Data are represented as mean ± SEM with *P<0.05.
(TIF)

S3 Fig Seizure severity is reduced after chronic induction of ER stress. Quantification of latency to stage 4 seizures, and the time spent between stage 4 to 5 seizures from 3-weeks old WT mice intraperitoneally injected with saline or Thapsigargin (Tg, 0.5 mg/kg) for 48 hours followed by injections with KA (60 mg/kg). The number of mice used in each condition is shown on the bottom of each bar. For the quantification, Student’s *t*-test was used. Data are represented as mean ± SEM with **P<0.01 and *P<0.05.
(TIF)

S4 Fig A time course of neural network activity and the number of active electrodes in MEAs. The spontaneous spike rates of WT cortical neuron cultures on MEA and the number of active electrodes at DIV 10–18 were recorded. The relative spontaneous spike rates are normalized to that from the same cultures during the 15-min recordings at DIV10. Data are represented as mean ± SEM (n = 6 cultures).
(TIF)

S5 Fig Drug treatments do not affect the number of active electrodes in MEA recordings. Quantification of active electrodes by comparing ‘after treatment’ to ‘before treatment’ of the same cultures during the 15-min recordings. A one-way ANOVA with Tukey test was used. Data are represented as mean ± SEM with ns: non-significant (n = 10–13 independent cultures).
(TIF)

S6 Fig Chronic induction of ER stress represses protein translation. Representative western blots of puromycin and Gapdh, and quantification of puromycin labeling in WT C57BL/6J (A) or *Mdm2*^*f/+*-*Emx1*-Cre-^ (B) cortical neuron cultures treated with vehicle (DMSO) or Tg for 4 hours with puromycin labeling occurring only during the fourth hour (n = 5 and 8 for A and B, respectively). Student’s *t*-test was used for data analysis. Data are represented as mean ± SEM with **P<0.01.
(TIF)

S7 Fig Mdm2-p53 signaling is no longer activated after 4-hour treatment of Thapsigargin in cortical neuron cultures. (A) Representative western blots of phospho (P)- Mdm2, Mdm2, and Gapdh from WT cortical neuron cultures treated with vehicle (DMSO) or Thapsigargin (Tg, 1 μM) for 4 hours (n = 6). (B) Representative western blots of Mdm2, Lamin A/C and HSP-90 after nuclear and cytosolic extraction using WT cortical neuron cultures treated with vehicle (DMSO) or Tg for 4 hours. Lamin A/C and HSP-90 serve as controls for nuclear and cytosolic fractions, respectively (n = 5). (C) Representative western blots of p53 and Gapdh from WT cortical neuron cultures treated with vehicle (DMSO) or Tg for 4 hours (n = 9). Student’s *t*-test was used for data analysis. Data are represented as mean ± SEM with **P<0.01, ns: non-significant.
(TIF)

S8 Fig ER stress response continues after 4-hour treatment of Thapsigargin in cortical neuron cultures. (A, B) Representative western blots of phospho (P)- eIF2α, eIF2α, and Gapdh from WT cortical neuron cultures treated with vehicle (DMSO) or Thapsigargin (Tg, 1 μM) for 1 hour (A) or 4 hours (B) (n = 8 and 11 for A and B, respectively). Student’s *t*-test was used for data analysis. Data are represented as mean ± SEM with *P<0.05, **P<0.01, ***P<0.001, ns: non-significant.
(TIF)

S9 Fig ER stress does not alter ribosome biogenesis. Quantitative real-time RT-PCR of 47S pre-rRNA normalized to Actin mRNA from *Mdm2*^*f/+*-*Emx1*-Cre-^ or *Mdm2*^*f/+*-*Emx1*-Cre+^ cortical neuron cultures treated with vehicle (DMSO) or Tg for 1 hour (n = 5 and 4 for *Mdm2*^*f/+*-*Emx1*-Cre-^ and *Mdm2*^*f/+*-*Emx1*-Cre+^, respectively). Student’s *t*-test was used for data analysis. Data are represented as mean ± SEM.
(TIF)

S10 Fig Knocking down p53 in *Mdm*^*2f/+-Emx1*-Cre+^ cortical neuron cultures restores ER stress-reduced neural network activity. (A) Quantification and representative western blots of p53 and Gapdh from WT cortical neuron cultures lentivirally transduced with a shRNA against p53 or a control non-target shRNA for 72 hours staring at DIV11 (n = 5). (B_1_) Raster plots of spontaneous spikes from representative 1-min recordings of *Mdm2*^*f/+*-*Emx1*-Cre+^ cortical neuron cultures lentivirally transduced with a shRNA against p53 or a control non-target shRNA for 72 hours staring at DIV11, followed by treatment with vehicle (DMSO) or Thapsigargin (Tg, 1 μM) for 1 hour at DIV14. (B_2_) Quantification of relative spontaneous spike rates by comparing ‘after treatment’ to ‘before treatment’ of the same cultures during the 15-min recordings. (C_1_) Representative average traces of spike amplitude of 1-min recording of *Mdm2*^*f/+*-*Emx1*-Cre+^ cortical neuron cultures lentivirally transduced with a shRNA against p53 or a control non-target shRNA for 72 hours staring at DIV11, followed by treatment with DMSO or Tg for 1 hour at DIV14. In the traces, the black lines represent the average of all the spikes within representative 1-min recordings. Traces are from the same designated electrodes before and after treatments. (C_2_) Quantification of average spontaneous spike amplitude by comparing ‘after treatment’ to ‘before treatment’ during the 15-min recordings of the same cultures. (D_1_) Representative traces of burst activity from *Mdm2*^*f/+*-*Emx1*-Cre+^ cortical neuron cultures lentivirally transduced with a shRNA against p53 or a control non-target shRNA for 72 hours staring at DIV11, followed by treatment with DMSO or Tg, 1 μM for 1 hour at DIV14. Traces are from the same designated electrode ‘before’ and ‘after’ drug treatments. (D_2_) Quantification of burst duration by comparing ‘after treatment’ to ‘before treatment’ during the 15-min recordings from the same cultures (n = 9 and 12 for cultures transduced with control shRNA and p53 shRNA, respectively). Student’s *t*-test was used for data analysis. Data are represented as mean ± SEM with *P<0.05, ns: non-significant.
(TIF)

S11 Fig Confirmation of Mdm2 knockdown efficiency in the cortex of *Mdm2*^*f/+-Emx1*-Cre+^ mice. Quantification and representative western blots of Mdm2 and Gapdh from cortex and cerebellum (non-cortex) regions of *Mdm2*^*f/+*-*Emx1*-Cre-^ or *Mdm2*^*f/+*-*Emx1*-Cre+^ mice (n = 10). Student’s *t*-test was used for data analysis. Data are represented as mean ± SEM with *P<0.05, ns: non-significant.
(TIF)

S1 Table Summary of mortality rate of mice after kainic acid-induced seizures. (DOCX)

S1 Appendix Summary of numerical data that underlies the graphs in each figure. (XLSX)
