# Supplementary figures and images for: Novel roles of ER stress in repressing neural activity and seizures through Mdm2- and p53-dependent protein translation
Source: PLoS Genet. 2019 Sep 26;15(9):e1008364. doi: 10.1371/journal.pgen.1008364 (PMC6762060; doi:10.1371/journal.pgen.1008364)

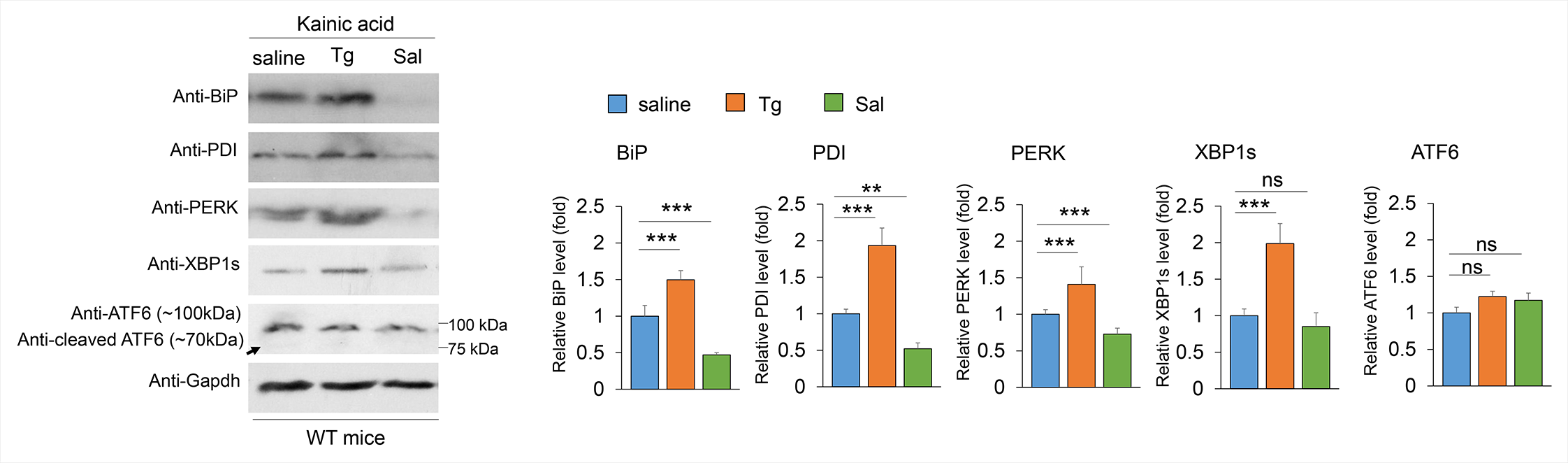

Supplement: S1 Fig — Quantification and representative western blots of BiP, PDI, PERK, XBP1s, ATF6 and Gapdh from total brain lysate of 3-week old WT mice intraperitoneally injected with saline, Thapsigargin (Tg, 2 mg/kg) or Salubrinal (Sal, 2 mg/kg) followed by kainic aicd (60 mg/kg). The arrow indicates the predicted position for cleaved ATF6. A one-way ANOVA with Tukey test was used. Data are represented as mean ± SEM with **P<0.01, ***P<0.001, ns: non-significant (n = 4). Of note, despite an elevation of XBP1s in Tg-treated groups, the mice treated with Salubrinal did not show reduction of XBP1s when compared to the mice treated with saline only. This is likely due to the fact that Salubrinal functions to inhibit eIF2α pathway and may not affect XBP1 splicing. (TIF) [file pgen.1008364.s001.tif]

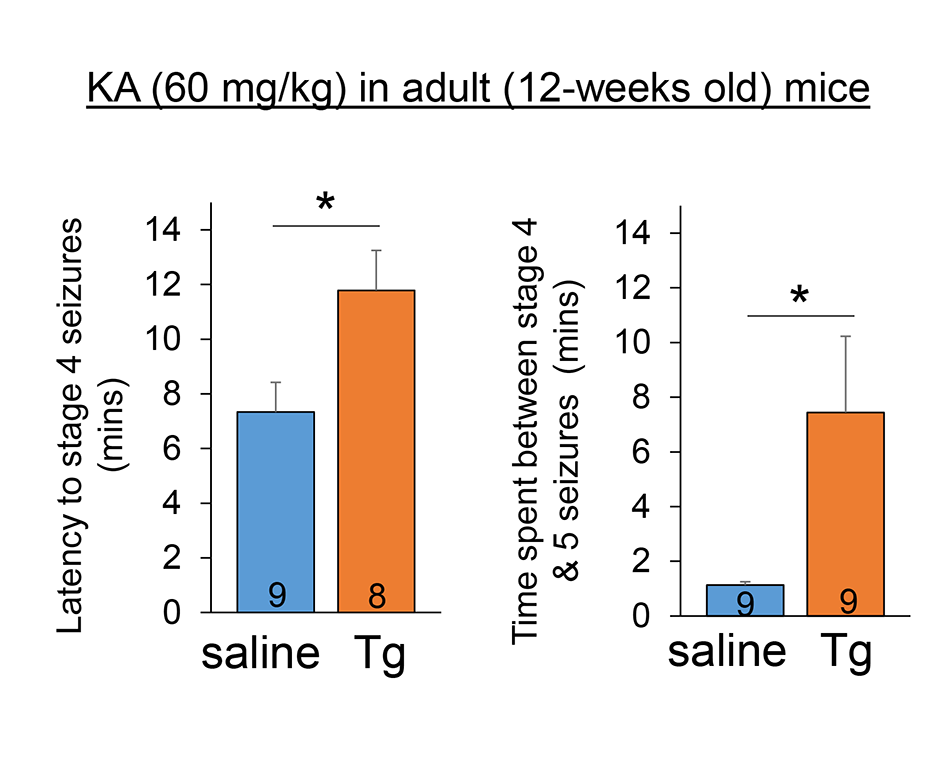

Supplement: S2 Fig — Quantification of latency to stage 4 seizures, and the time spent between stage 4 to 5 seizures from 12-weeks old WT mice intraperitoneally injected with saline or Thapsigargin (Tg, 2 mg/kg) for 3 hours followed by injections with KA (60 mg/kg). Injections of kainic acid with 30 mg/kg were not included in this experiment because preliminary tests showed no signs of stage 5 seizures in adult WT mice, likely because the resistance to KA-induced seizures is more apparent in adult mice of C57BL/6J background. The number of mice used in each condition is shown on the bottom of each bar. For the quantification, Student’s t-test was used. Data are represented as mean ± SEM with *P<0.05. (TIF) [file pgen.1008364.s002.tif]

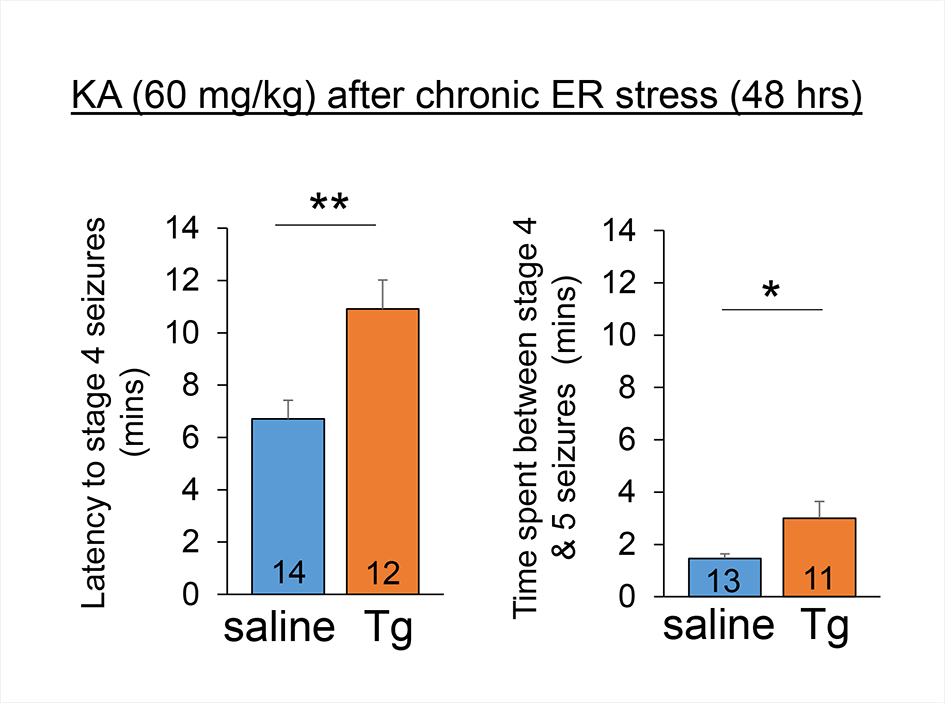

Supplement: S3 Fig — Quantification of latency to stage 4 seizures, and the time spent between stage 4 to 5 seizures from 3-weeks old WT mice intraperitoneally injected with saline or Thapsigargin (Tg, 0.5 mg/kg) for 48 hours followed by injections with KA (60 mg/kg). The number of mice used in each condition is shown on the bottom of each bar. For the quantification, Student’s t-test was used. Data are represented as mean ± SEM with **P<0.01 and *P<0.05. (TIF) [file pgen.1008364.s003.tif]

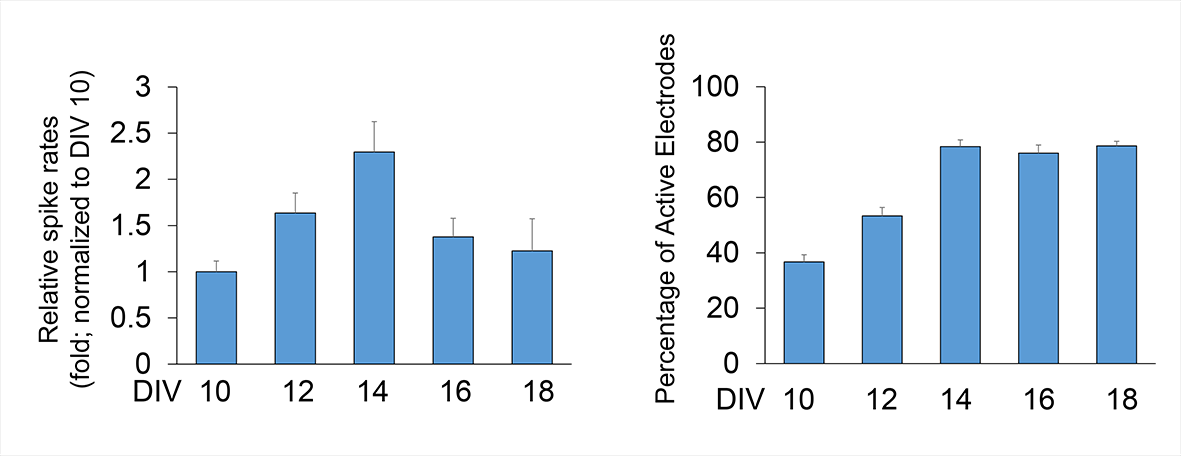

Supplement: S4 Fig — The spontaneous spike rates of WT cortical neuron cultures on MEA and the number of active electrodes at DIV 10–18 were recorded. The relative spontaneous spike rates are normalized to that from the same cultures during the 15-min recordings at DIV10. Data are represented as mean ± SEM (n = 6 cultures). (TIF) [file pgen.1008364.s004.tif]

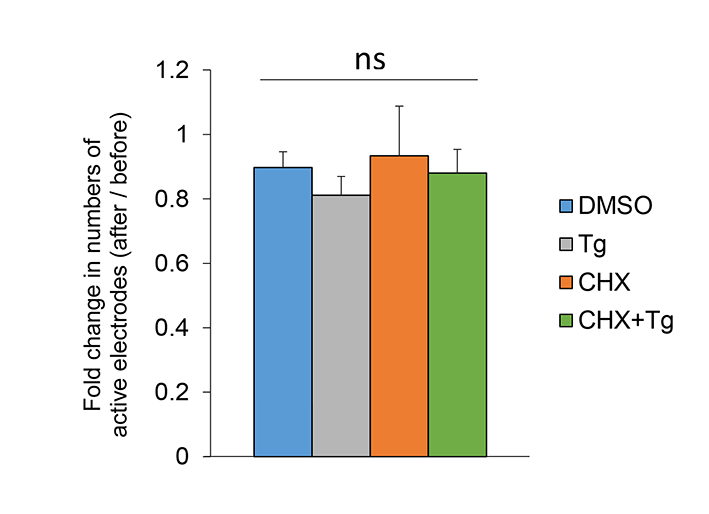

Supplement: S5 Fig — Quantification of active electrodes by comparing ‘after treatment’ to ‘before treatment’ of the same cultures during the 15-min recordings. A one-way ANOVA with Tukey test was used. Data are represented as mean ± SEM with ns: non-significant (n = 10–13 independent cultures). (TIF) [file pgen.1008364.s005.tif]

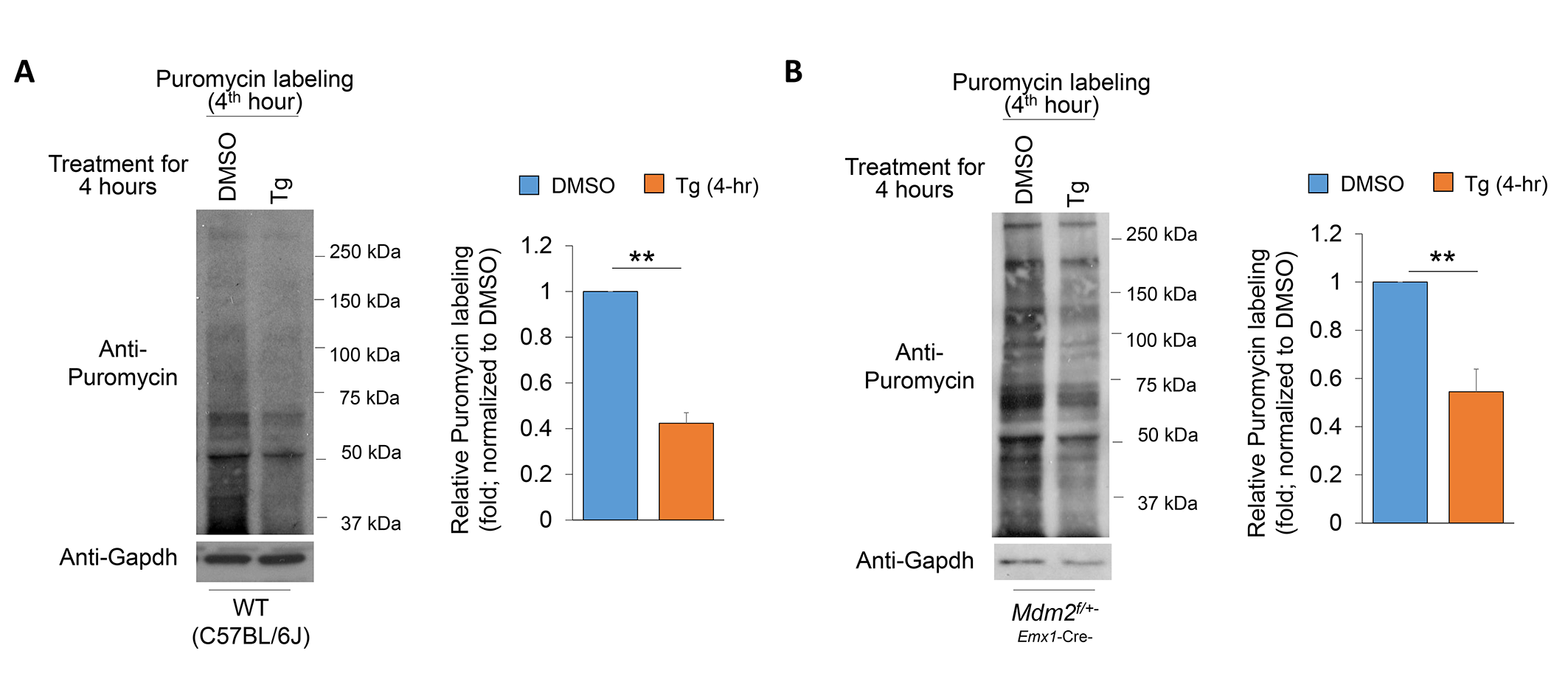

Supplement: S6 Fig — Representative western blots of puromycin and Gapdh, and quantification of puromycin labeling in WT C57BL/6J (A) or Mdm2f/+-Emx1-Cre- (B) cortical neuron cultures treated with vehicle (DMSO) or Tg for 4 hours with puromycin labeling occurring only during the fourth hour (n = 5 and 8 for A and B, respectively). Student’s t-test was used for data analysis. Data are represented as mean ± SEM with **P<0.01. (TIF) [file pgen.1008364.s006.tif]

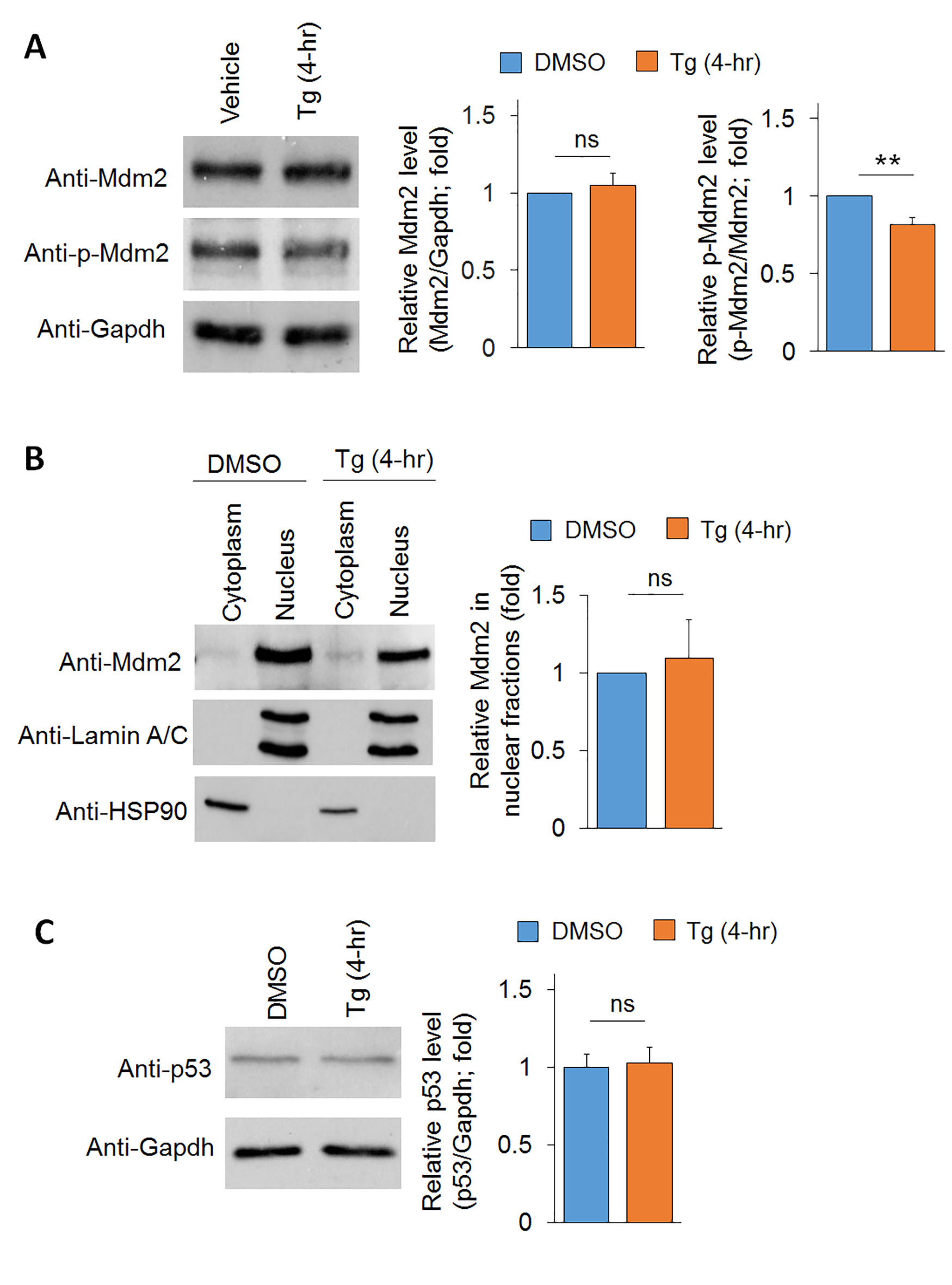

Supplement: S7 Fig — (A) Representative western blots of phospho (P)- Mdm2, Mdm2, and Gapdh from WT cortical neuron cultures treated with vehicle (DMSO) or Thapsigargin (Tg, 1 μM) for 4 hours (n = 6). (B) Representative western blots of Mdm2, Lamin A/C and HSP-90 after nuclear and cytosolic extraction using WT cortical neuron cultures treated with vehicle (DMSO) or Tg for 4 hours. Lamin A/C and HSP-90 serve as controls for nuclear and cytosolic fractions, respectively (n = 5). (C) Representative western blots of p53 and Gapdh from WT cortical neuron cultures treated with vehicle (DMSO) or Tg for 4 hours (n = 9). Student’s t-test was used for data analysis. Data are represented as mean ± SEM with **P<0.01, ns: non-significant. (TIF) [file pgen.1008364.s007.tif]

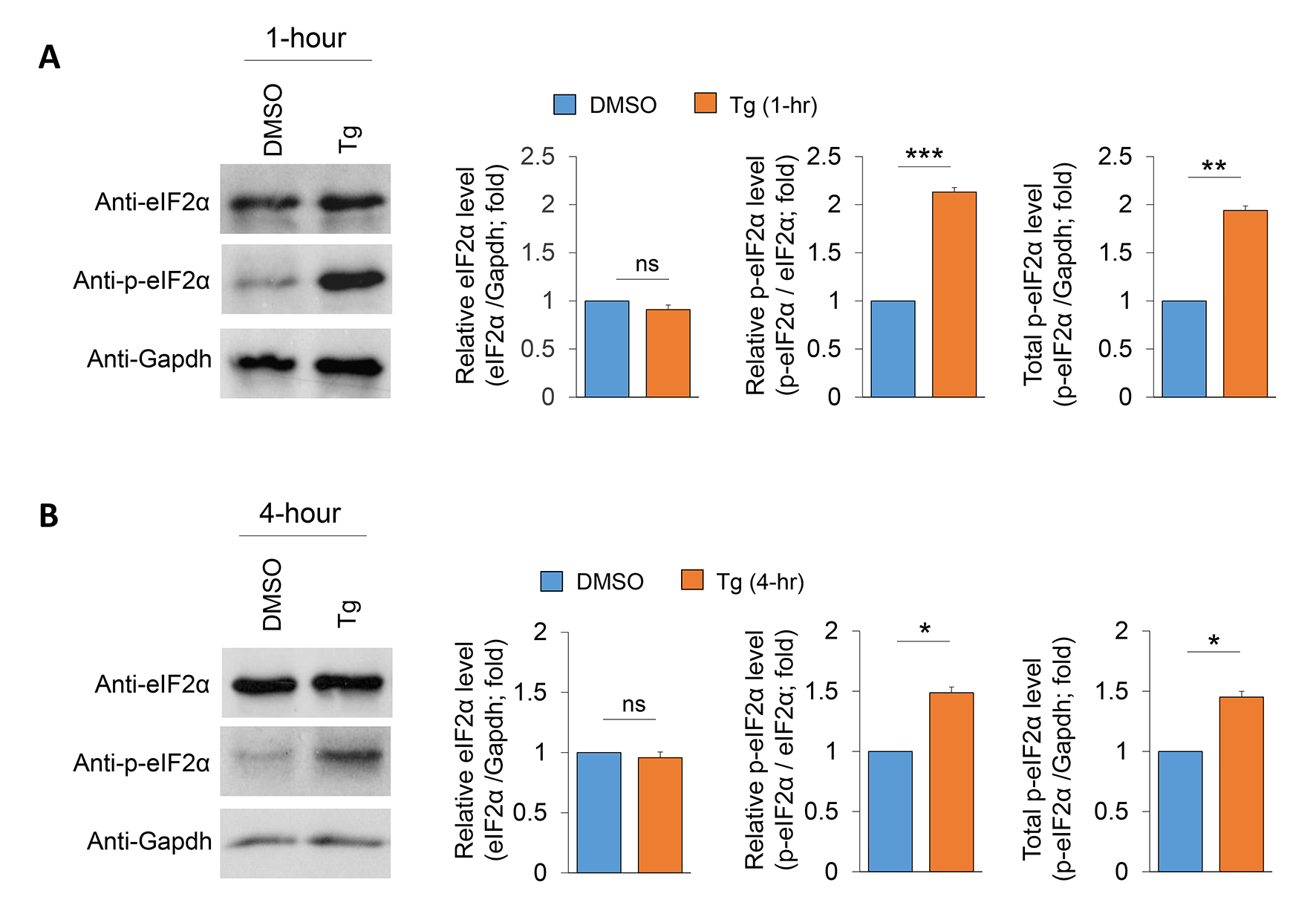

Supplement: S8 Fig — (A, B) Representative western blots of phospho (P)- eIF2α, eIF2α, and Gapdh from WT cortical neuron cultures treated with vehicle (DMSO) or Thapsigargin (Tg, 1 μM) for 1 hour (A) or 4 hours (B) (n = 8 and 11 for A and B, respectively). Student’s t-test was used for data analysis. Data are represented as mean ± SEM with *P<0.05, **P<0.01, ***P<0.001, ns: non-significant. (TIF) [file pgen.1008364.s008.tif]

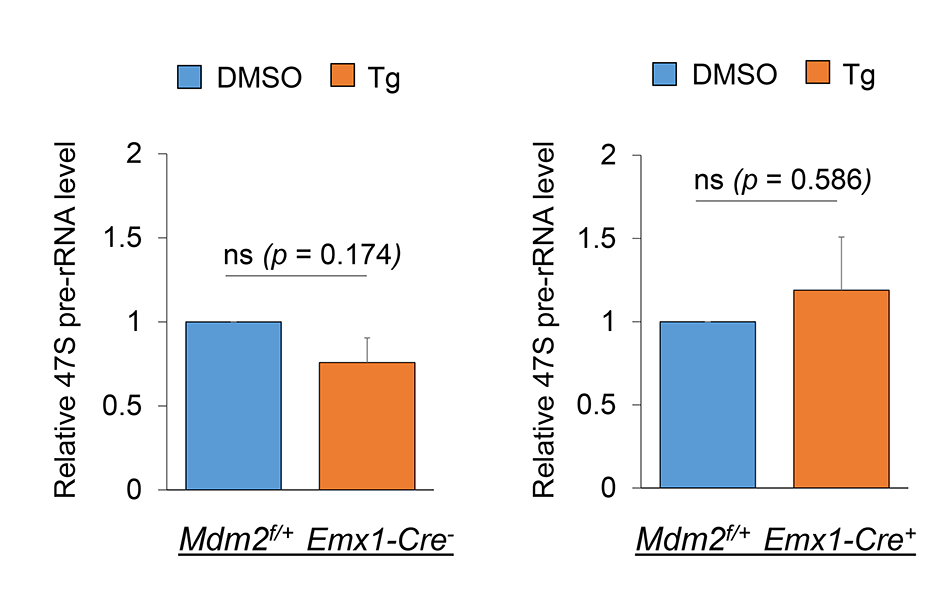

Supplement: S9 Fig — Quantitative real-time RT-PCR of 47S pre-rRNA normalized to Actin mRNA from Mdm2f/+-Emx1-Cre- or Mdm2f/+-Emx1-Cre+ cortical neuron cultures treated with vehicle (DMSO) or Tg for 1 hour (n = 5 and 4 for Mdm2f/+-Emx1-Cre- and Mdm2f/+-Emx1-Cre+, respectively). Student’s t-test was used for data analysis. Data are represented as mean ± SEM. (TIF) [file pgen.1008364.s009.tif]

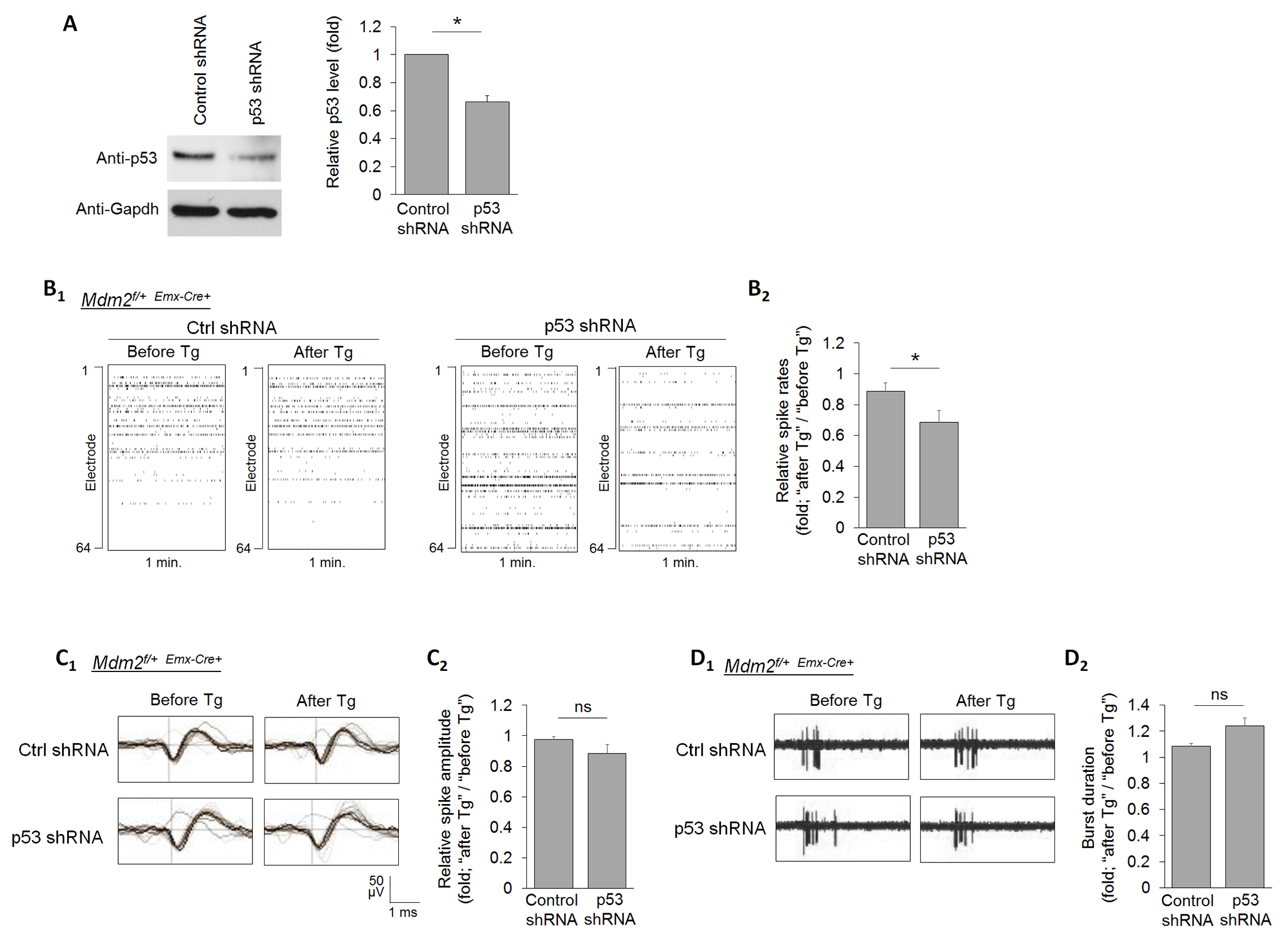

Supplement: S10 Fig — (A) Quantification and representative western blots of p53 and Gapdh from WT cortical neuron cultures lentivirally transduced with a shRNA against p53 or a control non-target shRNA for 72 hours staring at DIV11 (n = 5). (B1) Raster plots of spontaneous spikes from representative 1-min recordings of Mdm2f/+-Emx1-Cre+ cortical neuron cultures lentivirally transduced with a shRNA against p53 or a control non-target shRNA for 72 hours staring at DIV11, followed by treatment with vehicle (DMSO) or Thapsigargin (Tg, 1 μM) for 1 hour at DIV14. (B2) Quantification of relative spontaneous spike rates by comparing ‘after treatment’ to ‘before treatment’ of the same cultures during the 15-min recordings. (C1) Representative average traces of spike amplitude of 1-min recording of Mdm2f/+-Emx1-Cre+ cortical neuron cultures lentivirally transduced with a shRNA against p53 or a control non-target shRNA for 72 hours staring at DIV11, followed by treatment with DMSO or Tg for 1 hour at DIV14. In the traces, the black lines represent the average of all the spikes within representative 1-min recordings. Traces are from the same designated electrodes before and after treatments. (C2) Quantification of average spontaneous spike amplitude by comparing ‘after treatment’ to ‘before treatment’ during the 15-min recordings of the same cultures. (D1) Representative traces of burst activity from Mdm2f/+-Emx1-Cre+ cortical neuron cultures lentivirally transduced with a shRNA against p53 or a control non-target shRNA for 72 hours staring at DIV11, followed by treatment with DMSO or Tg, 1 μM for 1 hour at DIV14. Traces are from the same designated electrode ‘before’ and ‘after’ drug treatments. (D2) Quantification of burst duration by comparing ‘after treatment’ to ‘before treatment’ during the 15-min recordings from the same cultures (n = 9 and 12 for cultures transduced with control shRNA and p53 shRNA, respectively). Student’s t-test was used for data analysis. Data are represented as mean ± S [file pgen.1008364.s010.tif]

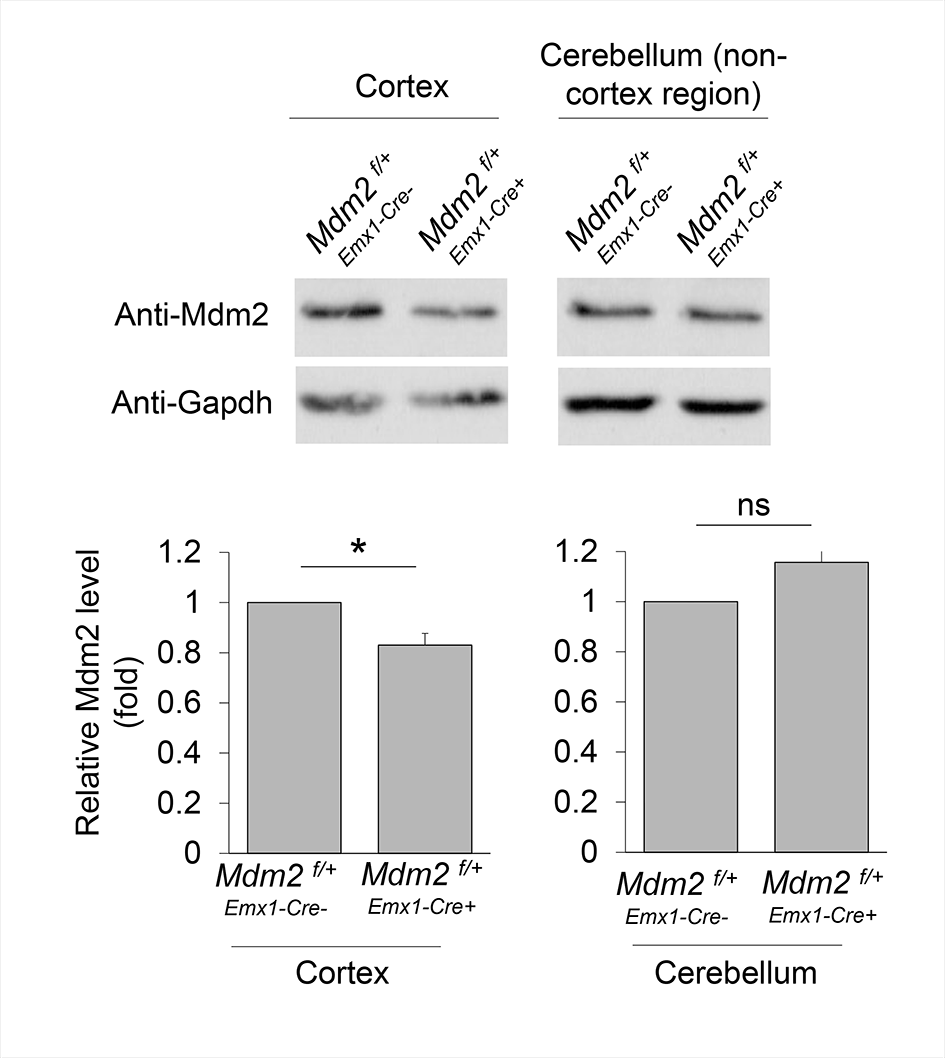

Supplement: S11 Fig — Quantification and representative western blots of Mdm2 and Gapdh from cortex and cerebellum (non-cortex) regions of Mdm2f/+-Emx1-Cre- or Mdm2f/+-Emx1-Cre+ mice (n = 10). Student’s t-test was used for data analysis. Data are represented as mean ± SEM with *P<0.05, ns: non-significant. (TIF) [file pgen.1008364.s011.tif]
