# Supplementary material for: Novel roles of ER stress in repressing neural activity and seizures through Mdm2- and p53-dependent protein translation
Source: PLoS Genet. 2019 Sep 26;15(9):e1008364. doi: 10.1371/journal.pgen.1008364 (PMC6762060; doi:10.1371/journal.pgen.1008364)
Supplement: S1 Table — (DOCX) [file pgen.1008364.s012.docx]

**S1 Table: Mortality rate after kainic acid-induced seizures in mice.**

| **Corresponding figures** | **Genotypes** | **Drug pre-treatment** | **Dosage of kainic acid** | **Mortality rate** |
| --- | --- | --- | --- | --- |
| Fig. 1B | WT (C57BL/6J) | Saline | 30 mg/kg | 80% (8/10) |
|  |  | Thapsigargin |  | 67% (6/9) |
|  |  | Saline | 60 mg/kg | 100% (8/8) |
|  |  | Thapsigargin |  | 100% (9/9) |
| Fig. 1C | WT (C57BL/6J) | Saline | 30 mg/kg | 91.7% (11/12) |
|  |  | Salubrinal |  | 83.3% (10/12) |
|  |  | Saline | 60 mg/kg | 100% (8/8) |
|  |  | Salubrinal |  | 100% (8/8) |
| Fig. 6A | Mdm2^f/+^-Emx1-Cre^-^ | Saline | 60 mg/kg | 88.9% (8/9) |
|  |  | Thapsigargin |  | 87.5% (7/8) |
|  |  | Pifithrin |  | 100% (7/7) |
|  |  | Thapsigargin+Pifithrin |  | 88.9% (8/9) |
| Fig. 6B | Mdm2^f/+^-Emx1-Cre^+^ | Saline | 60 mg/kg | 88.9% (8/9) |
|  |  | Thapsigargin |  | 88.9% (8/9) |
|  |  | Pifithrin |  | 100% (9/9) |
|  |  | Thapsigargin+Pifithrin |  | 90% (9/10) |
| S2 Fig. | WT (C57BL/6J) | Saline | 60 mg/kg | 100% (9/9) |
|  |  | Thapsigargin |  | 88.9% (8/9) |
| S3 Fig. | WT (C57BL/6J) | Saline (48 hr) | 60 mg/kg | 100% (14/14) |
|  |  | Thapsigargin (48 hr) |  | 100% (12/12) |
